# Structure of the G protein chaperone and guanine nucleotide exchange factor Ric-8A bound to Gαi1

**DOI:** 10.1038/s41467-020-14943-4

**Published:** 2020-02-26

**Authors:** Levi J. McClelland, Kaiming Zhang, Tung-Chung Mou, Jake Johnston, Cindee Yates-Hansen, Shanshan Li, Celestine J. Thomas, Tzanko I. Doukov, Sarah Triest, Alexandre Wohlkonig, Gregory G. Tall, Jan Steyaert, Wah Chiu, Stephen R. Sprang

**Affiliations:** 1 Center for Biomolecular Structure and Dynamics, University of Montana, Missoula, MT 59812 USA; 2 Department of Bioengineering and James H. Clark Center, Stanford University, Stanford, CA 94305 USA; 3 Division of Biological Sciences, University of Montana, Missoula, MT 59812 USA; 4 Macromolecular Crystallography Group, Stanford Synchrotron Radiation Light Source, SLAC National Accelerator Laboratory, Stanford University, Stanford, CA 94025 USA; 5 Structural Biology Brussels, Vrije Universiteit Brussel (VUB), Brussels, Belgium; 6 VIB-VUB Center for Structural Biology, VIB, Brussels, Belgium; 7 Department of Pharmacology, University of Michigan Medical School, Ann Arbor, MI 48109 USA; 8 Biosciences Division of CryoEM and Bioimaging, SSRL, SLAC National Accelerator Laboratory, Stanford University, Menlo Park, CA 94025 USA; 9 Graduate Program in Biochemistry and Biophysics, University of Montana, Missoula, MT 59812 USA; 10 Present Address: Regeneron Pharmaceutical, Inc., Tarrytown, NY USA

**Keywords:** GTP-binding protein regulators, Cryoelectron microscopy, X-ray crystallography

## Abstract

Ric-8A is a cytosolic Guanine Nucleotide exchange Factor (GEF) that activates heterotrimeric G protein alpha subunits (Gα) and serves as an essential Gα chaperone. Mechanisms by which Ric-8A catalyzes these activities, which are stimulated by Casein Kinase II phosphorylation, are unknown. We report the structure of the nanobody-stabilized complex of nucleotide-free Gα bound to phosphorylated Ric-8A at near atomic resolution by cryo-electron microscopy and X-ray crystallography. The mechanism of Ric-8A GEF activity differs considerably from that employed by G protein-coupled receptors at the plasma membrane. Ric-8A engages a specific conformation of Gα at multiple interfaces to form a complex that is stabilized by phosphorylation within a Ric-8A segment that connects two Gα binding sites. The C-terminus of Gα is ejected from its beta sheet core, thereby dismantling the GDP binding site. Ric-8A binds to the exposed Gα beta sheet and switch II to stabilize the nucleotide-free state of Gα.

## Abstract (web summary)

Ric-8A is guanine nucleotide exchange factor (GEF) that also acts as a folding chaperone for its Gα subunit. Here, the authors present the structure of Ric-8A bound to nucleotide-free Gαi1, revealing the mechanism by which Ric-8A exerts both GEF and chaperone activity.

## Introduction

Ric-8A is a cytosolic guanine nucleotide exchange factor (GEF) that activates heterotrimeric G protein alpha subunits (Gα)^1,2^. As a chaperone required for Gα biogenesis and membrane localization, Ric-8 homologs are essential to life in multicellular eukaryotes^3–5^. Genetic^6–8^ and biochemical data^9^ support a role for Ric-8A in G protein-coupled receptor (GPCR)-independent regulation of asymmetric cell division that is essential for embryonic development^10^. Ric-8A exhibits GEF and chaperone activity towards Gα of the i, q, and 12/13 classes^1^, while Ric-8B performs these functions for Gαs and Gαolf—each in a variety of cellular contexts^11,12^. Both GEF and chaperone activities are stimulated by Casein Kinase II phosphorylation^13^. That the Gα-class specificity of Ric-8A and Ric-8B is the same for both GEF and chaperone activities^14^ suggests a common mechanistic basis for the two activities.

Ric-8A adopts an armadillo (ARM)/HEAT repeat domain architecture and is structurally unrelated to GPCRs^15^. Biophysical data show that nucleotide-free Gαι1 is structurally dynamic when bound to Ric-8A^16,17^ and shares some properties with GPCR-bound Gα^18^. Namely, Ric-8A induces rotational dynamics in the Gα helical domain^19^ and binds to the C-terminus of Gα^16,20^. In contrast to GPCRs, there is evidence that Ric-8A forms extensive interactions with the Gα switch regions that undergo GTP-dependent conformational change^1,17,20–22^, and with the Gα β-sheet scaffold^17^. To better understand the structural basis of Ric-8A GEF and chaperone activity, we have determined the structure of the Ric-8A:Gαi1 complex, using both cryo-electron microscopy (cryo-EM) and X-ray crystallography.

## Results

### Cryo-EM and X-ray crystallographic analysis of Ric-8A:Gαi1

To form the complex we used the N-terminal 491-residue fragment of rat Ric-8A, which is a more active GEF than the full-length (530 amino acid) protein and, to reduce conformational heterogeneity, we used the N-terminal 31-residue truncation mutant of rat Gαi1 (Δ31NGαi1), an efficacious substrate for Ric-8A^16^. Recombinant Ric-8A(1–491) was phosphorylated by Casein Kinase II at S435 and T440, which is necessary and sufficient for stimulation of GEF activity^13^ (Supplementary Fig. 1). Hereafter we refer to phosphorylated Ric-8A(1–491) as Ric-8A and Δ31NGαi1 as Gα.

**Fig. 1:**
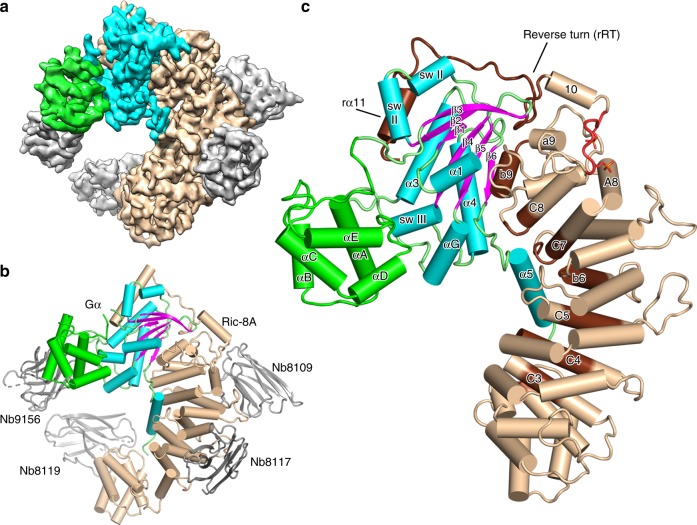
Architecture of the Ric-8A:Gα:4Nb complex. **a** Cryo-EM 3D map of Ric-8A:Gα:4Nb is shown with Ric-8A colored wheat, Gα GTPase and Helical domains colored cyan and green, respectively, and nanobodies colored gray. **b** Annotated ribbon and cylinder drawing, colored as in **a**, but with the β-strands of Gα rendered in magenta. **c** The Ric-8A:Gαi1:4Nb complex is shown with the nanobodies removed and the loop segments of the Gα GTPase domain colored green. Segments of Ric-8A that contact Gα are rendered in dark brown. Ric-8A residues 335–340, which include the two phosphorylation sites are rendered in red.

To stabilize and limit the dynamics of the Ric-8A:Gα complex for crystallographic and cryo-EM experiments, we developed a panel of camelid nanobodies (Nb)^23^ that specifically recognize either Gαi1, Ric-8A, or the complex of the two. We formed a series of Ric-8A:Gα:Nb complexes that included from one to four Nbs from this panel. The quality and resolution of cryo-EM reconstructions derived from these complexes was improved in step with the number of Nbs in the complex. We determined the cryo-EM structure of a complex of Ric-8A:Gα with three Nbs bound to Ric-8A and one bound to the helical domain of Gα. Together, these Nbs do not significantly affect Ric-8A GEF activity (Supplementary Fig. 2). The structure was determined from 327,493 particles with a sufficient orientation distribution derived from 8468 movie images, yielding a 3.9 Å resolution reconstruction (Fig. 1, Supplementary Figs. 3, 4, 5a and Supplementary Table 1).

We obtained crystals of Ric-8A:Gα bound to the three Ric-8A-specific Nbs used to generate the cryo-EM structure. Diffraction from these crystals was highly anisotropic (Supplementary Fig. 5b), extending to 4.6 Å along a*, and b*, and 3.3 Å along c*, affording measurement of a 90% complete anisotropic dataset (Supplementary Table 2). Initial crystallographic phases were determined by molecular replacement and subsequently used to fit the cryo-EM density map. Iterative cycles of model-building and refinement utilized both cryo-EM and X-ray diffraction data to generate the final models (Supplementary Table 1 and 2). In the following discussion, we use prefixes “r” and “g” for residue and secondary structure identifiers of Ric-8A and Gα, respectively. ARM/HEAT repeat helices of Ric-8A are designated according to their position in the repeat: “A”, “B” or “C” for ARM repeats or “a” and “b” for HEAT repeats, followed by the sequence number of the repeat (1 through 9). We use the established nomenclature for Gα secondary structure^2,24,25^. Descriptions of the crystal structure of Ric-8A:Gα refer to chains A and B, the better ordered of the two complexes in the asymmetric unit.

The cryo-EM reconstruction reveals Ric-8A residues 2–487 and the whole of Gα with the exception of the disordered linker (residues 50–76) between the Helical and GTPase domains (Fig. 1a, b and Supplementary Movie 1). The crystal structure of the Ric-8A:Gα complex also reveals continuous electron density for Ric-8A except for the linkage between 422 and 430. Notably, residues C-terminal to the last HEAT repeat (r430–r491) are disordered in structures of Ric-8A in which Gα is absent^15,20^. In the crystal structure, which lacks Nb9156, the helical domain of Gα and its connections to the GTPase domain, including much of gα1 and all of switch I, are disordered. Otherwise the cryo-EM and X-ray models of Ric-8A:Gα are in good agreement, although certain loop regions in both Gα and Ric-8A show significant divergence (Supplementary Fig. 6). Small angle X-ray scattering (SAXS) measurements of the complex are consistent with the X-ray and cryo-EM structures (Supplementary Fig. 7).

### Ric-8A displaces the Gα C-terminus and induces GDP release

**Fig. 2:**
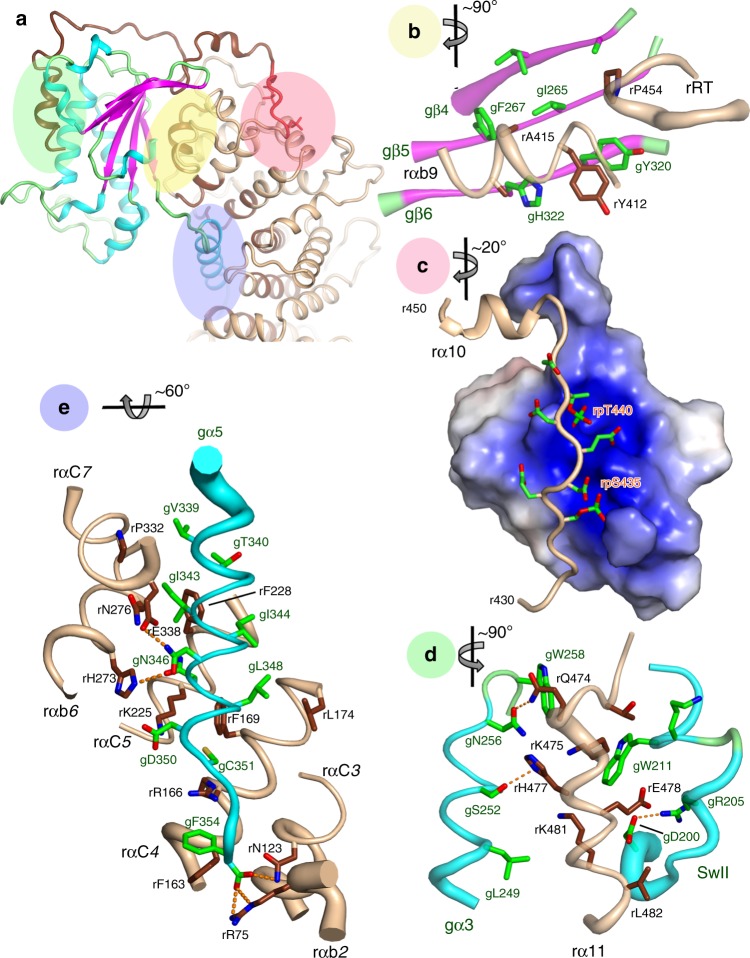
Interactions of Ric-8A with Gαi1. Three major Ric-8A:Gα contact surfaces and phosphorylation sites are highlighted in the green, yellow, pink, and blue overlays (panel **a**) and enlarged in panels **b**–**e**. Axes adjacent to each panel label indicate the rotation applied to the overall schematic to generate the panel view. **b** Interaction between Gα β-sheet and the Ric-8A C-terminal ARM/HEAT repeat helix rαbA9 and reverse turn r451–r457 (rRT) from the crystal structure. Polypeptide backbones are rendered as tubes, with diameter proportional to *B*-factor at Cα. Carbon atoms of Ric-8A and Gαi1 are rendered in deep brown, and green, respectively, and oxygen and nitrogen atoms are, respectively, colored red and blue. **c** Acidic Ric-8A peptide with phosphorylated residues rpS335 and rpT440 bound to the positively charged surface formed by the 8th and 9th Ric-8A ARM/HEAT repeats from the crystal structure. **d** Interaction of rα11 with gα3 and Switch II from cryo-EM. Putative hydrogen bonds (donor–acceptor contact < 3.5 Å) are depicted with orange dashed lines. **e** Contacts between gα5 and residues in successive ARM/HEAT repeats of Ric-8A, from the crystal structure.

**Fig. 3:**
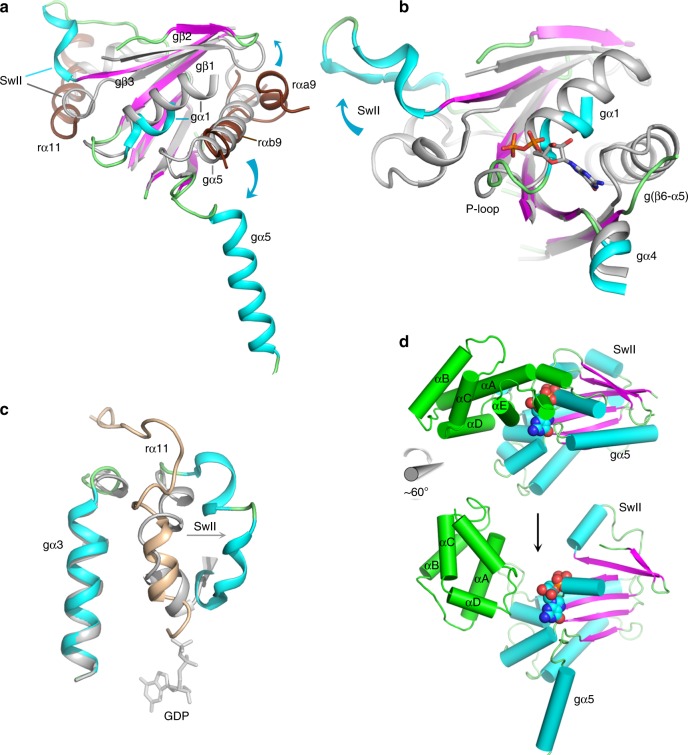
Conformational changes induced in Gα by its interaction with Ric-8A. In panels **a**–**c**, GDP•Pi-bound Gαi1 (PDB ID 1GIT) [10.2210/pdb1GIT/pdb], rendered in gray is superimposed on the crystal structure of Ric-8A-bound Gα using the Cα atoms of Gαi1 residues 219–225 (gβ4), 262–270 (gβ5), and 318-325 (gβ6). **a** Conformational changes due to binding of Ric-8A αa9, αb9, and RT to Gαi1. **b** Ric-8A-induced conformation changes dismantle the Gα nucleotide-binding site (see text). GDP from 1GIT is included as a stick model for reference. **c** Displacement of Switch II by rα*11*. The position of GDP bound to Gαi1•GDP is shown as a stick model. **d** Ric-8A-induced rotation of the helical domain away from the GTPase domain of Gα: top, Gαi1•GDP (1GIT) rendered with helices as cylinders and β-strands as ribbons and the helical domain colored green; atoms of GDP are rendered as spheres. Bottom, the cryo-EM-derived model of Ric-8A-bound Gα with GDP from 1GIT shown as a reference point.

Binding of Ric-8A to the GTPase domain of Gα at three non-contiguous contact surfaces bury more than 3200 Å^2^ of solvent-accessible surface area (Fig. 2a). Together, these interactions destabilize the guanine nucleotide-binding site (Fig. 3). At the core of the complex, Ric-8A αb9 (r411–r415) and the reverse turn r451–r457 (rRT) interact with the gβ4-γβ6 strands of Gα (Fig. 2b and Supplementary Fig. 8a and b).

**Fig. 4:**
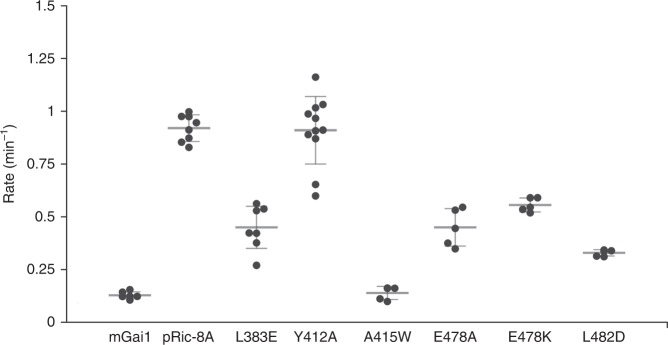
Mutational analysis of selected residues at the Ric-8A:Gα interface. GDP–GTP exchange rates were measured by the rate of tryptophan fluorescence increase upon addition of ΔN31Gαi1 to Ric-8A and GTPγS at final concentrations of 2 μM Ric-8A, 1 μM ΔN31Gαi1 and 10 μM GTPγS. Student’s two-tailed *t*-test probabilities that mutant Ric-8A nucleotide exchange rates fall within the distribution of wild-type Ric-8A (*n* = 8) are: L383E, *n* = 7, *p* = 1 × 10^−6^; Y412A, *n* = 11, *p* = 0.83 (not significant); A415W, *n* = 4, *p* = 7 × 10^−12^; E478A, *n* = 5, *p* = 1.7 × 10^−5^; E478K, *n* = 5, *p* = 2.9 × 10^−8^; L482D, *n* = 4, *p* = 6 × 10^−9^. mGai1 represents the intrinsic exchange rate for myristoylated Gαi1 (*n* = 5, *p* = 5 × 10^−10^). Horizontal bars represent means and 1 standard deviation.

Ric-8 αb9 occupies the site of gα5 in GDP-bound Gα, and consequently gα5 is ejected from the concave surface of the Gα β-sheet (Fig. 3a). Ric-8A residues rY412, rA415, rA416, and rL418 in rαb9 substitute for nonpolar residues of gα5, to stabilize the hydrophobic surface of the Gα β-sheet core. These residues are conserved in both A and B isoforms of vertebrate Ric-8 (Supplementary Fig. 9a), and form Van der Waals interactions with residues in gβ4–gβ6 that are conserved in both Gαi and Gαs families (Supplementary Fig. 9b). The rW415A mutation severely impairs GEF activity (Fig. 4), although the Y412A mutation does not. At the same interface, steric interactions with rαa9 lever the antiparallel gβ2–gβ3 hairpin away from the GTPase core (Fig. 3a). As a unit, gβ1–gβ5 undergo a ~5° counter-clockwise rotation as viewed from the concave surface of the Gα β-sheet. Changes in the orientation of gβ1–gβ3, in particular, destabilize and induce partial disorder in gα1. This, as described below, triggers separation of the Helical and GTPase domains of Gα.

The ~90° rotation of α5 away from the GTPase domain core (Fig. 3a) reconfigures the “TATC” motif in the g(β6-α5) GDP purine-binding loop (Fig. 3b). This structural change perturbs the conserved NKKD motif (in gβ4-αG) that confers specificity for guanosine nucleotides^26^. Displacement of these two loops dismantles the GDP purine-binding site. The hydrophobic contact between the N-terminus of gα4 and the Ric-8A αB8-αC8 turn (Supplementary Fig. 10) is also important, as indicated by the impairment of GEF activity resulting from the rL383E mutation (Fig. 4).

### Binding of the Gα C-terminus to the Ric-8A ARM repeat trough

After its ejection from the Gα β-sheet, gα5 is accommodated in a broad trough formed by helices rαb2 through rαB8 that line the concave surface of ARM/HEAT superhelix of Ric-8A (Fig. 2e and Supplementary Fig. 8g and h), as observed also in the structure of Ric-8A bound to the C-terminus of transducin^20^. Indeed, after superposition of the respective Ric-8A models, the main-chain and side-chain atoms of the C-termini of transducin (residues 334–350) and Gαi1(338–354) align with an RMS deviation of 0.36 Å. The predominantly hydrophobic amino acid residues of gα5 that interact with the Ric-8A trough (e.g. gF336, gV339, gI343, gI344, and gL348) otherwise pack against the GTPase domain β-sheet of nucleotide-bound Gα^27^. Most of the Ric-8A residues that contact gα5 are conserved in Ric-8B (Supplementary Fig. 9a), whereas several of the Ric-8A-contacting residues in gα5 are not conserved among Gα classes (Supplementary Fig. 9b).

### Phosphorylation of Ric-8A stabilizes its interface with Gα

Ric-8A phosphorylation acts as an entropic clamp to promote GEF activity. The Ric-8A αb9 and RT segments that interact with the Gα β-sheet are connected by an intrinsically disordered sequence, r430–r450^15^ followed by rα10. The segment spanning r430–r440 is rich in acidic amino acids, and binds within a basic groove formed by rαA8 (r344–r358) and rαa9 (r401–r410) (Fig. 2c). Phosphorylated rpS435 and rpT440, which are well-ordered (Supplementary Fig. 8i–l) and form multiple ion-pair interactions with conserved lysine and arginine side chains in the Ric-8A electropositive groove, help to immobilize the r430–r450 connector and consequently stabilize rαb9 and rRT that interact with gβ4–gβ6. Accordingly, we found earlier that charge → neutral mutations of rR345Q and rK349A, which interact with rpS435 and adjacent acidic residues, reduce both basal and phosphorylation-stimulated GEF activity of Ric-8A^15^.

### Ric-8A interactions with Gα Switch II and α3

Ric-8A helix α11 (r471–r491), which is preceded by an elongated peptide (r458–r470) that forms an extended arch over the α3-β5 loop of Gα, packs between Gα Switch II and gα3 (Fig. 2d, and Supplementary Fig 8c–f). In the GTP-bound state of Gα, these two elements form the Gα effector protein-binding site^28^. The ejection of gα5 from the Gα β-sheet together with interactions between α11 and the Switch II and gα3 interfaces was recently deduced from steered molecular dynamics calculations consistent with small angle X-ray scattering and crosslinking data^22^. rα11 occupies the position of Switch II in the G protein heterotrimer^29^, and also in Gαi1 bound to GTP analogs^27^ and in the Gαi1•GDP•Pi product complex^30^ (Fig. 3c). However, Switch II is disordered in Gαi1•GDP and probably also in nucleotide-free Gα^25^. Ric-8A interactions with switch II are functionally important, since mutations of two Gα-contacting residues in rα11, rE478, and L482, which are conserved in both Ric-8A isoforms, impair GEF activity (Fig. 4). The conformations of Switch II observed in the X-ray and cryo-EM structures differ from each other, and from those adopted in nucleotide-bound Gα^28^ (Supplementary Fig. 8e, f). Remodeling of Switch II may be facilitated by Ric-8A-induced displacement of β2-β3 (Fig. 3a) described above.

### Ric-8A-induced reorientation of the Gα helical domain

Displacement of gα5 and reorientation of gβ2-gβ3 eliminates a nexus of stabilizing interactions with the C-terminal residues of gα1, which become disordered (Fig. 3a). As a consequence, contacts between the GTPase and helical domains of Gα are disrupted. As observed in the cryo-EM structure, the helical domain undergoes a counter-clockwise rotation of ~60° around an axis roughly aligned with αD of the Gα helical domain (Fig. 3d). By this motion, a channel opens between the helical and GTPase domains, providing a path of egress for the nucleotide. The magnitude of the rotation is less than that observed in crystal and cryo-EM structures of GPCR:G protein complexes^18,31^, or deduced from double electron–electron resonance studies of Ric-8A:Gα^19^, possibly because it is limited by steric interactions between the Nbs bound to Gα and Ric-8A. The variability in the orientation of the helical domain is also suggested by normal-mode analysis of the SAXS data (Supplementary Fig. 7). The coupled interactions between Ric-8A and Gαi1, described above, induce profound conformational changes that dismantle the Gα guanine nucleotide-binding site (Fig. 3b and Supplementary Movie 2).

## Discussion

The basis for the Gα class selectivity of Ric-8 isoforms is not readily apparent. The great majority of residues at the Gα contact residues are conserved in Ric-8A and Ric-8B (Supplementary Fig 9a). Gα residues that interact with rαB9 and rRT are conserved in the Gαi and Gαs classes. However, several residues in gα5 differ between Gαs and Gαi classes (Supplementary Fig. 9b), and, as suggested^20^, the latter may engage in more productive interactions with Ric-8A than their Gαs counterparts.

The structure of the complex suggests mechanisms that produce the chaperone activity of Ric-8A, by which it promotes folding and stabilization of nucleotide-free Gα^3^. It is remarkable that the magnitude of conformational changes induced in Gα by Ric-8A far exceed those sufficient to effect GDP release in GPCR–G protein complexes^2,18^. Comparison of the Ric-8A:Gα complex with that of the Gαi2:β_1_γ_2_ heterotrimer bound to the A_1_ adenosine receptor^32^ (Supplementary Fig. 11) shows that both GEFs induce conformational changes or disorder within the Gα P-loop and in gα1. Binding within the transmembrane cavity of GPCRs, the C-terminus of Gα (gα5) undergoes a 60° rotation and 5 Å displacement^18^, thereby inducing rearrangement of gβ6-gα5 and, to a lesser extent, gβ4-αG, both of which are purine recognition elements. The destabilization of gα1 by loss of contacts with gα5 is transmitted both to the P-loop and the hinge between the Ras and helical domains, permitting the release of contacts between the two^2^. By its wholesale ejection of gα5 from the Gα β-sheet and perturbation of the Gα β-sheet itself, which is not observed in interactions with GPCRs, Ric-8A produces the same outcome. Remarkably, the C-terminus of Gα, which tends to disorder, forms anchoring contacts with both GPCRs and with Ric-8A. In contrast, GPCRs do not induce reorientation of the gβ2–gβ3 β-hairpin, β1, or major structural changes in Switch II that are observed in the complex of Ric-8A with Gαi1.

The large interaction surface formed by Ric-8A with Switch II and the Gα β-sheet core may be particularly related to its role as a chaperone. Ric-8A stabilizes nucleotide-free Gα subunits in the absence of Gβγ^16^. Molecular dynamics simulations suggest that contacts between gα5 and the Gα β-sheet are dynamic in nucleotide-free Gα^33^. Interactions with Ric-8A would shield these hydrophobic surfaces from exposure in the cytosol. Switch II is also a dynamic structure, even in the GDP-bound state of Gα^26^ and would likewise be stabilized by Ric-8A α11. Ric-8A(1–491) partially rescues Gαi1 biosynthesis in *Ric-8A*^*−/−*^ cells, but not that of Gαq, for which full-length Ric-8A is required^34^. Hence, the C-terminal ~40 residues of Ric-8A, which harbor three of the five CKII phosphorylation sites^13^, must play a critical role in Ric-8A chaperone activity. Nevertheless, it is clear that the mechanism by which Ric-8A stabilizes dynamic regions of the nucleotide-free Gα GTPase domain also underlie its GEF activity.

**Fig. 5:**
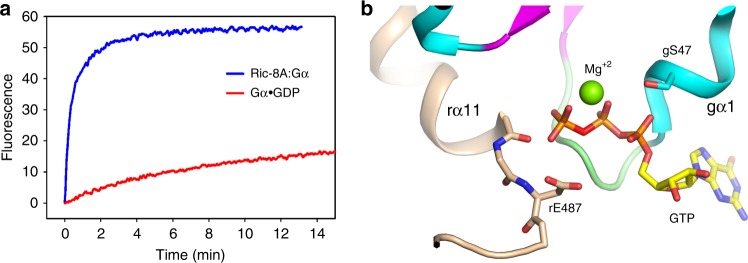
The P-loop of Gαi1 provides a pre-ordered binding site for GTP in the complex with Ric-8A. **a** Progress curve for GTP (10 μM) binding to purified Ric-8A:Gαi1 complex (1 μM) (red), relative to that for nucleotide exchange at Gαi1•GDP (1 μM). **b** Superposition of a model of GTP (PDB ID 1CIP) [10.2210/pdb1CIP/pdb] onto the nucleotide-binding site of Ric-8A-bound Gαi1. Serine 47 of gα1 is positioned for Mg^2+^ coordination (see text).

The extensive interface between Ric-8A and Gα prompts the question how the two proteins are able to dissociate upon binding of GTP to Gα, a process that is kinetically facile in comparison to the overall exchange reaction (Fig. 5a and Supplementary Fig. 2). Although displaced slightly by steric conflict with rα11 (Fig. 3b), the conformation of the P-loop is largely retained in the nucleotide-free Gα bound to Ric-8A (Fig. 5b), thus providing a preformed platform for subsequent binding of GTP. Electrostatic repulsion between the C-terminus of r11 and the γ-phosphate of GTP as modeled in Fig. 5b, could promote its release from its binding site between gα3 and Switch II, allowing Switch II to refold into its native GTP-bound conformation. Disruption of Switch II interactions with Ric-8A would restore the native structure of gβ2–gβ3, promote its interaction with gα1 and thereby destabilize the remaining interface of Ric-8A with the GTPase domain. The dynamics that accompany Ric-8A binding to Gα•GDP and subsequent release of Gα•GTP remain to be explored.

## Methods

### Preparation of crosslinked Ric-8A and Gαi1

Bis-sulphosuccinimidyl suberate (BS3, Pierce-Thermo-Fisher Scientific) was dissolved in water to a concentration of 100 μM. K100 reagent (CovalX) is provided as a 2 mg/ml aqueous solution and contained a proprietary mixture of inert carbon chain spacers (lengths between 8.8 and 13.2 Å), separating 1-hydroxyl-7-azabenzotriozole groups. Ric-8A:Gαi1 complex, prepared as described^16,17,19^ was dialyzed into 20 mM phosphate buffered saline (PBS) pH 7.4, 1 mM DTT, and final concentration adjusted to 20 μM in the same buffer. For each 100 μl of complex, 20 μl of K100 (2 mg/ml) and 25 μl of BS3 (100 μM) was added, incubated at room temperature for 30 min and the reaction quenched by addition of 10 μl of 1 M Tris pH 8.5. Samples were centrifuged at 14,000 rpm on a desk-top centrifuge for 10 min at 4 °C, to remove particulate matter, eluted though a tandem 5 ml HiTrap desalting column (GE Healthcare) to remove excess cross-linking reagents. Proteins were eluted in 20 mM PBS, concentrated to 0.3 mg/ml and 1 mM fresh DTT added as the final step. Cross-linked samples were analyzed by Coomassie-stained SDS–PAGE and verified by Western blotting using an anti-Ric-8A monoclonal antibody^3^ and mouse anti-Γαi1 monocolonal antibody (Enzo Lifesciences). The crosslinked preparation contained cross-linked Ric-8A:Gαi1, free Gαi1, and Ric-8A in roughly equal proportion.

### Development of Camelid Nbs

Llamas (*Lama glama*) were immunized with the cross-linked Ric-8A:Gαi1 preparation. Peripheral blood lymphocytes were isolated from the immunized animals for extraction of total RNA, generation of cDNA and PCR amplification to isolate cDNAs encoding heavy chain variable domains (VHH) for subcloning into the pCTCON2 vector for display on the cell surface of *Saccharomyces cerevisiae* EBY100 cells, as described^23,35,36^. Cells expressing Nbs that selectively recognize Gαi1, Ric-8A, or the crosslinked Ric-8A:Gαi1 were identified by three-color FACS sorting on a FACS AriaIII (BD Biosciences), using Dylight 505 (Pierce, Thermo Scientific)-conjugated Ric-8A, Dylight 488-conjugated Gαi1, and R-phycoerythrin goat anti-mouse antibody (Lucron) to label nanobody-expressing clones. Three rounds of selection were conducted using 10 μM fluorescent proteins in 20 mM PBS pH 7.4, 100 mM NaCl, and 2 mM DTT. Nanobody sequences from selected clones yielding Nbs that exclusively bind to Gαi1, to Ric-8A either free or bound to Gαi1, or exclusively to the Ric-8A:Gαi1 complex were subcloned in a pMESy4 vector for periplasmic expression in *E. coli* WK6 cells as described^37^. Nanobody sequences encode a C-terminal hexahistidine affinity tag.

Of the set of 27 Nbs discovered in screening that show strong expression, 20 were capable of binding to either free Ric-8A or to Ric-8A in complex with Gαi1 but not to Gαi1, three bound exclusively to the Ric-8A:Gαi1 complex and three bound to Gαi1 or its complex with Ric-8A but not to Ric-8A alone. From this set was identified a set of four Nbs capable of binding simultaneously to Ric-8A:Gαi1, as determined by co-elution as a stable hexameric complex from a GE Healthcare HiLoad 16/600 Superdex 200 pg column and validated by mass spectrometry on a MicroFlex MALDI-ToF MS (Brucker Microflex). Of this set, Nb8109, Nb8117, and Nb8119 bind to Ric-8A and the Ric-8A:Gαi1 complex and Nb9156 binds to Gαi1.

### Nanobody expression and purification

Nb 8109, 8117, 8119, and 9156 were encoded in pMESy4 vectors for periplasmic expression in WK6 *Escherichia coli* cells^23^. Briefly, overnight cultures were grown in 50 ml LB media containing 100 μg/ml Ampicillin, 100 mM Glucose, and 1 mM MgCl_2_ in shaker flasks at 190 rpm and 37 °C. After ~16 h cells were pelleted at 4000 rpm (2200 × *g*) for 10 min using a benchtop Sorvall Legend RT. Re-suspended pellets were added to 1 L TB media containing 100 μg/ml ampicillin, 5 mM glucose, and 1 mM MgCl_2_ then incubated at 190 rpm and 37 °C until achieving an OD_600_ of 0.7–1.0, at which point temperature was lowered to 28 °C and growths were induced with 300 μM isopropyl β-d-1-thiogalactopyranoside (IPTG). Approximately 16 h post-induction, cells were pelleted at 8000 rpm (12,000 × *g*) for 15 min in a Sorvall RC 6+ Centrifuge and cell pellets were stored at −80 °C. Pellets were re-suspended in 100 ml of TES buffer (0.2 M Tris pH 8, 0.5 mM EDTA, 0.5 M sucrose) at 4 °C, vortexed and allowed to stir for a minimum of 1 h. Cells were then added dropwise to a 200 ml solution of TES/4 buffer (0.05 M Tris pH 8, 0.125 mM EDTA, 0.125 M sucrose), then stirred for 1 h at 4 °C. Lysate was then spun down at 8000 rpm (12,000 × *g*) for 30 min and supernatant was loaded on a gravity column with Ni-NTA agarose resin (Qiagen). Nanobody-bound resin was washed with 20 column volumes of Wash 1 (0.05 M Tris pH 8, 1 M NaCl), 5 column volumes of Wash 2 (0.05 M MES pH 6, 1 M NaCl), 10 column volumes of Wash 3 (0.05 M Tris pH 8, 0.5 M NaCl), and then eluted with 3 column volumes of elution buffer (0.05 M Tris pH 8, 0.2 M NaCl, 0.5 M imidazole). Eluted nanobody was dialyzed overnight in 0.05 M Tris pH 8 and 0.2 M NaCl, then concentrated to 5–10 mg/mL before use or storage at −80 °C. Before use all Nb were gel purified using a Superdex 200 10/300 GL size-exclusion column (GE Healthcare) in gel filtration buffer: 50 mM HEPES pH 8, 150 mM NaCl, and 1 mM TCEP.

Complexes of Ric-8A, ΔN31Gαi1, and Nb Nb8109, Nb8117, Nb8119 in the presence or absence of Nb9156 were formed by incubating Ric-8A, ΔN31Gαi1, and Nb at a 1:2:2 (per nanobody) molar ratio on ice for 16 h and subsequently purified by gel filtration chromatography using a GE Healthcare HiLoad 16/600 Superdex 200 pg in gel filtration buffer.

### Gα and Ric-8A protein expression and purification

N-terminally glutathione-S-transferase-tagged Rat Gαi1 with a 31 residue N-terminal truncation (∆N31Gαi1) was expressed from a pDest15 vector in Bl21(DE3) RIPL *Escherichia coli* and purified as previously described^16,38^. N-terminally hexahistidine-tagged Rat Ric-8A (residues 1–491, hereafter Ric-8A) was expressed and purified as previously described in a pET28a vector with BL21(DE3) RIPL *Escherichia coli* cells^15–17^. Following removal of the hexahistidine tag using Tobacco Etch Virus protease, Ric-8A protein was loaded onto a Source 15Q column and eluted with a 500 mM NaCl gradient at 180 mM NaCl and subsequently loaded on a GE Healthcare HiLoad 16/600 Superdex 200 column for size-exclusion chromatography in gel filtration buffer containing 50 mM Tris pH 8, 150 mM NaCl, and 1 mM Tris(2-carboxyethyl)phosphine (TCEP).

Ric-8A was phosphorylated using casein kinase II (New England Biolabs) as previously described^15,39^. Briefly, purified Ric-8A was mixed 1:1 with a 2X kinase reaction buffer containing 0.1 M HEPES pH 8, 0.1 M NaCl, 20 mM MgCl_2_, 2 mM EGTA, and 1 mM DTT. ATP was added to a final concentration of 5 mM. 160 Units of casein kinase II were added per mg of Ric-8A, and the reaction was allowed to proceed for ~16 h at room temperature. Phosphorylated protein was loaded onto a Source 15Q column pre-equilibrated with 50 mM HEPES pH 8, 25 mM NaCl, and 2 mM β-ME, and eluted from a 500 mM NaCl gradient at 210 mM NaCl. Phosphorylated Ric-8A exhibits an ~2 mS/cm shift compared to un-phosphorylated Ric-8A (Supplementary Fig. 1b). All procedures described hereinafter were conducted with phosphorylated Ric-8A comprising residues 1–491 of the full-length protein and referred to as Ric-8A. Myristoylated Gαi1 (mGαi1), used in nucleotide exchange assays, was prepared as described previously^15^. Mutants of Ric-8A, used in nucleotide exchange assays, were constructed via the QuikChange II XL Site-Directed Mutagenesis Kit (Agilent). Expression, purification, and phosphorylation proceeded as described above.

### Guanine nucleotide exchange assays

Assays were conducted by measuring the change in Gαi1 tryptophan fluorescence in the presence or absence of Ric-8A using either ΔN31Gαi1 or mGαi1, as described^17^. All proteins were buffer exchanged and assays were conducted in 50 mM HEPES pH 8, 150 mM NaCl, 10 mM MgCl_2_, 1 mM TCEP. Experiments were carried out at 20 °C using a LS55 luminescence spectrometer (Perkin Elmer) with 4 nm slit widths (excitation 295 nm, emission 345 nm). GEF activity for Ric-8A and Ric-8A mutants were measured by preincubating 450 μl of Ric-8A and GTPγS in a quartz fluorescent cuvette prior to addition of 50 μl of 10 μM mGαi1 for a final concentration of 0.5 μM Ric-8A, 1 μM mGαi1, and 10 μM guanosine 5′-O-[gamma-thio]triphosphate (GTPγS) in 500 μl. GTPγS binding to size-exclusion chromatography purified complexes of Ric-8A: ΔN31Gαi1 with and without Nb were compared to intrinsic nucleotide exchange of ΔN31Gαi1. GTPγS was added to preincubated complexes for final concentrations of 1 μM complex (or ΔN31Gαi1) and 10 μM GTPγS. For each assay, a minimum of five technical replicates were taken for each sample; more replicates were performed if the series of assays was conducted over several days, to control for changes in sample activity. Progress curves were fit to single or double (If a slow binding phase was detected) exponential rate models using SigmaPlot 7. Statistical significance of rate differences between reference and test samples was determined by a two-tailed Student’s *t*-test. Probability that differences are derived from a random distribution is reported. All data points are shown in box plots that show mean and standard deviation for each data set^40^.

### Crystallization of the ΔN31Gαi1:Ric-8A:3Nb complex

Crystallization trials for the Ric-8A:ΔN31Gαi1:Nb8109:Nb8117:Nb8119 complex were conducted by vapor diffusion using commercially available crystallization screening kits. Sitting drops were set on 96-2 well INTELLI-PLATEs (Art Robbins Instruments) using a Gryphon crystallization robot (Art Robbins Instruments) at 3–10 mg/mL protein complex at a 1:1 v/v ratio with precipitation solution. Initial crystallization conditions were identified from hits on the ShotGun screen (MD1-88 Molecular Dimensions). Further optimization was carried out by grid screening variations in sodium malonate (Hampton Research) concentration and pH and by crystal seeding by hanging drop on 24-well VDXm plates (Hampton Research). Crystal seed stocks were prepared with the Seed Bead kit (Hampton Research) in 1.4 M sodium malonate pH 6.9. 0.9 μl of protein stock was added to 0.6 μl of reservoir and 0.3 μl of crystal seed stock and incubated at 12 °C for a minimum of 1–2 weeks. Optimal crystals were obtained from hanging drops containing 3.6 mg/ml Ric-8A:ΔN31Gαi1:3Nb, 1.4 M sodium malonate pH 6.9 at 12 °C.

### Crystallographic data collection and processing

Diffraction data were measured at the NSLS-II FXS beamline from LN_2_ cryoprotected crystals measuring ~50 μm × 50 μm × 5 μm employing a helical data collection mode^41^, using X-rays of 0.9793 wavelength. A Si (111) double crystal monochromator was used to focus the X-ray beam to a spot size of 1 μm × 1 μm at the sample. Data were measured in the Phi axis rotation mode in 0.2° fames, at 0.06 s/frame at a beam attenuation of 0.7. Diffraction data were measured on an Eiger 16M detector at a 133 Hz frame rate. Due to significant (>0.5 Å) differences in unit cell axis dimension between crystals, data from a single crystal were selected for data processing and structure determination. In view of the considerable anisotropy of diffraction (~4.6 Å along a* and b* and 3.3 Å along c*), three separate data-processing strategies were conducted using the AutoPROC v1.0.2 software toolbox^42^ for evaluation in subsequent model-building and refinement steps. All three utilized XDS^43^ for data indexing and initial integration. The Standard Isotropic protocol uses SCALEA and TRUNCATE from the CCP4^44^ to generate isotropic data with a resolution cutoff (4.6 Å) determined by the criteria *R*_pim_ ≥ 0.6, *I*/*σ*(*I*) ≥ 2.0 and CC_1/2_ ≥ 0.3. The Extended Isotropic protocol employed POINTLESS and AIMLESS scaling and analysis software^45^ to generate scaled intensities extending to the 3.2 Å resolution with *I*/*σ*(*I*) ≥ 1.2. The Anisotropic Filtering protocol implemented in STARANISO^46^ generated a dataset that incorporates intensities within a locally averaged value of *I*/*σ*(*I*) to define an anisotropic diffraction cut-off surface (Supplementary Table 2).

### Crystallographic model building and refinement

All crystallographic calculations were conducted using the PHENIX 1.1.6 software package^47^ unless otherwise noted. Initial crystallographic phases for the Ric-8A:ΔN31Gαi1:3NB complex were determined by Molecular Replacement using atomic coordinates of Ric-8A (residues 1–426; PDB ID:6NMG), the Ras domain of Gαi1:GDP (PDB ID: 1BOF), and an anti-VGLUT nanobody (PDB ID:5OCL) as search models. An initial atomic model, consisting of coordinates for Ric-8A residues 1–426, residues 34–65 and 190–320 of ΔN31Gαi1 and three nanobody backbone models were manually refit into a sigma-weighted 2mFo-DFc map using Coot v0.8.6^48^ and refined using phenix.refine. Completion and refinement of the crystallographic model followed the following general strategy: (1) model-fitting to the cryo-EM reconstruction in regions of the structure in which the path of the polypeptide chain was not defined in (or differed substantially from) the 2mFo-DFc map followed by real space refinement with secondary structure and geometry restraints using phenix.real_space_refine^49^; (2) refitting of the latter to the 2mFo-DFc map and subsequent refinement; (3) refinement of the cryo-EM map (see below) using the crystallographic model as an alignment reference. In the regions in which the polypeptide path observed in the cryo-EM-derived and crystallographic models diverged, no attempt was made to bring them into agreement. Initial cycles of crystallographic model building and refinement were carried out using Standard Isotropic or Extended isotropic data sets derived from datasets derived from merging and scaling data from the two most strongly diffracting crystals. The final rounds of fitting and refinement of the complete model (excluding the C-terminus of Gα1, the Helical Domain, and Switch I, residues 51–184) utilized an Anisotropic Supplementary set comprising data from the single crystal that afforded the strongest diffraction and optimal merging statistics (see Supplementary Table 2). A TLS model was applied during refinement using phenix.refine. Model quality and correlation with the refined electron density were performed using MolProbity^50^. The atomic coordinates for the refined crystallographic model for the Ric-8A:ΔΝ31Gαi1:3Nb complex and associated structure factors are deposited in the RCSB Protein Data Bank^51^ (PDB ID 6TYL). Figures depicting atomic models were rendered using PyMol version 2.3 (Schrodinger, LLC)

### Cryo-EM data collection

Three microliters of the Ric-8A:ΔΝ31Gαi1:4Nb complex at 0.4 mg/ml with 0.01% NP40 were applied onto glow-discharged 200-mesh R2/1 Quantifoil grids (Electron Microscopy Sciences). Grids were blotted for 4 s and rapidly cryocooled in liquid ethane using a Vitrobot Mark IV (Thermo Fisher Scientific) at room temperature and 100% humidity. The samples were screened using a Talos Arctica cryo-electron microscope (Thermo Fisher Scientific) operated at 200 kV and then imaged in a Titan Krios cryo-electron microscope (Thermo Fisher Scientific) with GIF energy filter (Gatan) at a magnification of 130,000× (corresponding to a calibrated sampling of 1.06 Å per pixel). Micrographs were recorded using EPU software (Thermo Fisher Scientific) with a Gatan K2 Summit direct electron detector, where each image is composed of 30 individual frames with an exposure time of 6 s and a dose rate of 11.5 electrons per second per Å^2^. A total of 8670 movie stacks were collected with a defocus range of −1 to −3 μm.

### Cryo-EM image processing

All micrographs were motion-corrected using MotionCor2^52^ and the contrast transfer function (CTF) was determined using CTFFIND4^53^. All particles were autopicked using the NeuralNet option in EMAN2 v 2.31 and further checked manually, yielding 768,736 particles from selected 8468 micrographs. Particle coordinates were then imported to Relion v3.0.6, wherein multiple rounds of 2D classification were performed to remove poor 2D class averages. Meanwhile, ~20,000 particle images were selected to build an initial model using the “ab-initio 3D” program in cryoSPARC v2^54^. A total of 676,130 particles were used for 3D classification in Relion to remove the poor classes. Next, five rounds of 3D heterogeneous refinement were performed in cryoSPARC to further remove the bad particles. The final 3D refinement with 327,493 particles was performed in cryoSPARC using the “non-Uniform refinement” option with a soft mask of the complex density and a 3.85 Å resolution map was obtained. The resolution of the final map was estimated according to the 0.143 FSC criterion. A 3.9 Å low-pass filter was done to the final 3D map for better display (see more information in Supplementary Fig. 2 and Table 1).

### Cryo-EM model building

The cryo-EM model for the Ric-8A:ΔΝ31Gαi1:4Nbs complex was constructed with a refined crystallographic Ric-8A:ΔΝ31Gαi:3Nb model (in which only the GTPase domain was modeled) and a model of the helical domain of Gαi1 in complex with Nb9156 from the crystal structure of Gαi:Nb9156 complex (unpublished data). The final Ric-8A:ΔΝ31Gαi1:4Nb cryo-EM model was optimized by refinement using phenix.real_space_refine with secondary structure and geometry restraints^49^. Model quality was evaluated with MolProbity^50^.

### Small angle X-ray scattering

SAXS was performed at BioCAT beamline 18ID at the Advanced Photon Source, Chicago, with in-line size exclusion chromatography (SEC-SAXS) to separate sample from aggregates and other contaminants. Protein sample (~5 mg/ml in 50 mM HEPES, pH 8.0, 150 mM NaCl and 1 mM TCEP) was loaded onto a Superdex 200 Increase 10/300 GL column, which was run at 0.7 ml/min in an AKTA Pure FPLC (GE Healthcare Life Sciences). After detection by an in-line UV monitor, the sample passed through the SAXS flow cell, a 1.5 mm ID quartz capillary with 10 µm walls. Scattering intensity was recorded using a Pilatus3 1M (Dectris) detector which was placed 3.67 m from the sample affording access to a range of momentum transfer (*q*) from 0.0065 to 0.35 Å^−1^ [*q* = 4π sin(*θ*)/*λ*]. Exposures of 0.5 s were acquired every 2 s during elution. Data were reduced using BioXTAS RAW v1.6.0^55^. Buffer blanks were created by averaging regions flanking the elution peak and subtracted from exposures selected from the elution peak to create the *I*(*q*) vs. *q* curves used for subsequent analyses. Ab initio molecular envelopes were computed by the ATSAS v2.6.2 package^56^ programs DAMMIN^57^. Ten bead models were reconstructed in DAMMIF^58^, which were aligned and averaged in DAMAVER^59^. The Molecular envelope was visualized, and atomic models fit to molecular envelopes using Chimera v1.10.2^60^. The conformational flexibility of Ric-8A:ΔN31Gαi1 was modeled by coarse-grained fitting with respect to experimental SAXS data using SREFLEX program in the ATSAS software package^56^. Normal mode analysis was conducted with automatic determination of rigid body units. The final disposition of rigid body units after application of normal mode projections was determined by rigid body refinement with respect to the computed SAXS profile^61^. CRYSOL software from the ATSAS software package was used to model scattering profiles from atomic coordinates.

Amino acid sequence alignments were conducted using Clustal Omega^62^ via its web-server (https://www.ebi.ac.uk/Tools/msa/clustalo/).

### Reporting summary

Further information on research design is available in the Nature Research Reporting Summary linked to this article.

## Supplementary information

Supplementary Information

Peer Review

Reporting Summary

Description of Additional Supplementary Files

Supplementary Movie 1

Supplementary Movie 2

## Data Availability

Coordinates of Ric-8A:Δ31Gαi1:4Nb model from cryo-EM are deposited in the RCSB Protein Data Bank (PDB) with ID 6UKT[10.2210/pdb6UKT/pdb]. Coordinates of Ric-8A:Δ31Gαi1:3Nb model from crystal structure are deposited in the PDB with ID 6YTL[10.2210/pdb6YTL/pdb]. The cryo-EM reconstruction is deposited in the Electron Microscopy Data Bank with id EMD20812[https://www.ebi.ac.uk/pdbe/entry/emdb/EMD-20812]. The small angle X-ray scattering data for the Ric-8A:Δ31Gαi1 complex is deposited in the Small Angle Scattering Biological Database (SASBDB)^63^ with accession code SASDG95[https://www.sasbdb.org/data/SASDG95]. The source data underlying Fig. 4 and Supplementary Fig. 2a are provided as a Source Data file. All other relevant data are available from the authors upon reasonable request.

## Source Data

Source Data
