# Supplementary material for: Structure of the G protein chaperone and guanine nucleotide exchange factor Ric-8A bound to Gαi1
Source: Nat Commun. 2020 Feb 26;11:1077. doi: 10.1038/s41467-020-14943-4 (PMC7044438; doi:10.1038/s41467-020-14943-4)
Supplement: Supplementary file 1 — Supplementary Information [file 41467_2020_14943_MOESM1_ESM.pdf]

Supplementary Data

Structure of the cytosolic G protein alpha chaperone and guanine nucleotide exchange factor Ric-8A bound to Gαi1

McClelland, *et al.*

Supplementary Table 1
Cryo-EM Data Collection and Refinement Statistics

**Data collection and processing**	Ric-8A(491):Δ31NGαi1:4Nb
Microscope	Titan Krios
Voltage (kV)	300
Camera	Gatan K2 Summit
Pixel size (Å)	1.06
Total Dose (e-/Å^2)	69
Defocus range (μm)	-1 to -3
Number of micrographs	8,670
Number of initial particles	768,736
Symmetry	C1
Number of final particles	327,493
Resolution (0.143 FSC, Å)	3.90
**Atomic model refinement**	
Software	phenix.real_space_refine
Clashscore, all atoms	8.79
Poor rotamers (%)	0.57
Favored rotamers (%)	99.43
Ramachandran favored (%)	84.24
Ramachandran allowed (%)	15.76
MolProbity score	2.15
Bond RMSD (Å)	0.006
Angle RMSD (Å)	1.005

Supplementary Table 2
Crystallographic Data Collection and Refinement Statistics

**Data collection**	Ric-8A(491):Δ31NGαi1:3NB		
Software	AutoPROC/STARANISO	XDS/AIMLESS	SCALA
Reflection scaling method#	Anisotropic filtered (extended)	Isotropic (extended)	Isotropic (standard)
Wavelength (Å)	0.979	0.979	0.979
Resolution range (Å)*	39.58 - 3.3 (3.9 - 3.3) [a*, b*=4.6, c*=3.3]	39.65 - 3.3 (3.9 - 3.3)	39.65 - 4.6 (4.7 - 4.6)
Space group	$P\ 2_1$	$P\ 2_1$	$P\ 2_1$
Unit cell dimensions			
a, b, c (Å)	93.0 144.7 114.4	93.0 144.7 114.4	93.0 144.7 114.4
β (°)	94.7	94.7	94.7
Total reflections*	73843 (3449)	160159 (62218)	58730 (2930)
Unique reflections*	20707 (1035)	44840 (17203)	16296 (796)
Redundancy*	3.6 (3.3)	3.6 (3.6)	3.6 (3.7)
Completeness [spherical] (%)*	45.8 (6.0)	99.1 (99.1)	99.1 (99.5)
Completeness [ellipsoidal] (%)*	90.1 (58.2)	98.3 (91.5)	
Mean I/σ (I)*	3.7 (1.7)	1.9 (0.3)	4.1 (2.2)
Wilson B-factor	91.88		
R_{meas} †,*	0.21 (0.70)	0.55 (4.65)	0.18 (0.54)
$R_{p.i.m.}$ †,*	0.11 (0.38)	0.29 (2.42)	0.10 (0.28)
$CC_{1/2}$ †	0.99 (0.70)	0.97 (0.17)	0.99 (0.86)
**Refinement**			
R_{work} †,*	0.248 (0.319)		
R_{free} †,*	0.287 (0.323)		
CC_{work} †*	0.907 (0.719)		
CC_{free} †*	0.866 (0.893)		
Number of total atoms			
protein	15938		
total protein residues	2021		
RMS deviations			
bond lengths (Å)	0.002		
bond angles (°)	0.55		
Ramachandran favored (%)††	89.16		
Ramachandran allowed (%)††	10.53		
Ramachandran outliers (%)††	0.30		
Rotomer outliers (%)††	0.0		
Clash Score††	10.32		
Average B-factor			
Macromolecules	109.97		
Number of TLS groups	1		

The standard isotropic scaling method uses the classical isotropic treatment in truncate program where the resolution cut is determined by (1) Rpim ≤ 0.6. average I/σ(I) in a resolution bin ≥ 2.0, and $CC_{1/2} \geq 0.3$. The extended isotropic method includes all the measurements within the sphere of the highest observed resolution limit where the local [I/σ(I) ≥ 1.2; the extended anisotropic method uses the anisotropically-filtered reflections by STARANISO with the same resolution limit as the extended isotropic method. * Data for highest resolution shell are given in brackets. † $R_{meas}=\sum_{hkl}(n/n-1)^{1/2}\sum_i |I_i(hkl)-<I(hkl)>\rangle| / \sum_{hkl}\sum_i I_i(hkl)$, where $I_i(hkl)$ is the *i*th observation of the intensity of the reflection *hkl* and $<I_{hkl}>$ is the mean over *n* observations. $R_{p.i.m.}=\sum_{hkl}(1/n-1)^{1/2}\sum_i |I_i(hkl)-<I(hkl)>| / \sum_{hkl}\sum_i I_i(hkl)$. § $R_{work}=\sum_{hkl} || F_{obs}|-|F_{calc}|| / \sum_{hkl} |F_{obs}|$, where F_{obs} and F_{calc} are the observed and calculated structure-factor amplitudes for each reflection *hkl*. R_{free} was calculated for 5% of the diffraction data that were selected randomly and excluded from refinement. Correlation coefficients: $CC = \Sigma_i((x_i - <x>)(y_i - <y>))/(\Sigma_i((x_i - <x>)^2(y_i - <y>)^2)^{1/2}$, where x_i and y_i are the *i*th of *n* observations of quantities whose mean values are $<x>$ and $<y>$; for $CC_{1/2}$, x_i, and y_i correspond to intensity measurements derived from each of two randomly selected half-data sets from the set of unmerged data; For CC_{work} and CC_{free} x_i and y_i refer to observed structure factor amplitudes and structure factor amplitudes computed from the refined atomic model, respectively, for the working data set and the set used to compute R_{free}. ††Calculated using MolProbity

a

```
NB_8109  AQVQLQESGGGLEQAGDSLRLSCAASGLIVSNYAMGWFRQAPGKEREFVAYINWNGGVTY  60
NB_9156  -QVQLQESGGGLEQAGDSLRLSCAASGLIVSNYAMGWFRQAPGKEREFVAYINWNGGVTY  59
NB_8117  -QVQLQESGGGLVQPGGSLRLSCAASGIIFRSNGMAWYRQAPGKEREWVASITSFGDA-I  58
NB_8119  -QVQLQESGGGLVQAGGSLRLSCAASGGIVHISSMGWFRQAPGKQRELVATSPSNGDI-R  58
          *********** * *.********** *.   .*.*:******:** **     *.

NB_8109  YTNSVKGRFTISRDNAKNTVYLQMNSLKPEDTAVYYCARTSRASVTTRVADFGYWGQGTQ  120
NB_9156  YTNSVKGRFTISRDNAKNTVYLQMNSLKPEDTAVYYCARTSRASVTTRVADFGYWGQGTQ  119
NB_8117  YRDSVKGRFTISRDNARNAVSLQTNSLKTEDTAVYYCNTYPV---------NSAWGQGTQ  109
NB_8119  YADSVKGRFTLSRDNAKNTVSLQMNSLEPEDTAVYYCHSFLRHTAS--ASYNNYYGQGTQ  116
         * :*******:*****:*:* ** ***: ********                . :*****

NB_8109  VTVSSHHHHHHEPEA 135
NB_9156  VTVSSHHHHHHEPEA 134
NB_8117  VTVSSHHHHHHEPEA 124
NB_8119  VTVSSHHHHHHEPEA 131
         ***************
```

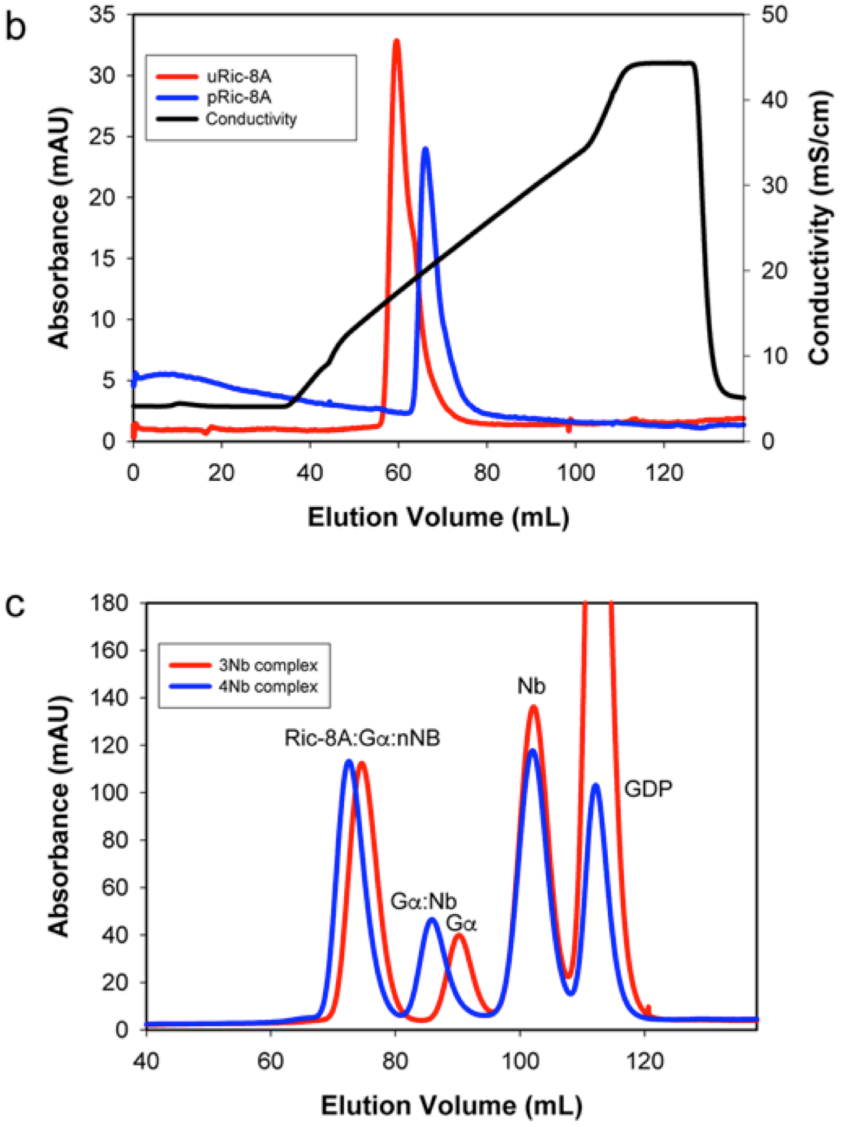

Supplementary Figure 1. Purification of Ric-8A:Gα:Nb complexes. **a**, Amino acid sequences of nanobodies used to form complexes. **b**, Anion exchange chromatography of phosphorylated (pRic-8A) and unphosphorylated (uRic-8A). **c**, Gel filtration of complexes at the following molar stoichiometric ratios: 1 Ric-8A:2 Gα:2 Nb 8109:2 Nb 8117:2 Nb 8119 (*red)* and 1 Ric-8A:2 Gα:2 Nb 8109:2 Nb 8117:2 Nb 8119:4 Nb 9156 (*blue*) molar stoichiometric ratios, Ric-8A and Gα correspond to the 1-491 residue construct of Ric-8A and the 31N-terminal truncation mutant of Gαi1, respectively, as described in the main text. (see Methods).

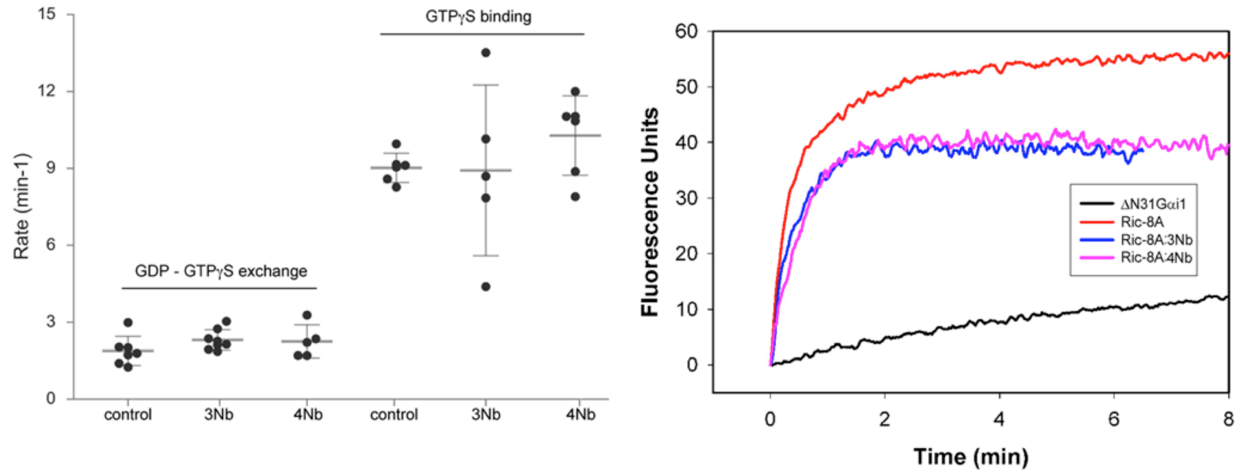

Supplementary Figure 2 Effect of nanobodies on GEF activity of Ric-8A. a, GDP-GTP exchange rates were measured by the rate of tryptophan fluorescence increase upon addition of ΔN31Gαi1 to Ric-8A and GTPγS at final concentrations of 2μM Ric-8A, 1μM ΔN31Gαi1 and 10μM GTPγS in the absence (control) or presence of Nb 8109 (4 μM), Nb 8117 (4 μM), Nb 8119 (4 μM) (3Nb) or the latter with the addition of (4 μM) Nb 9156 (4Nb). GTP binding rates were measured by tryptophan fluorescence increase upon addition of GTPγS (10 μM final concentration) to size exclusion chromatography-purified Ric-8A:ΔN31Gαi1:nNb (1 μM). Progress curves for the 3Nb and 4Nb GDP-GTPγS exchange and 4Nb GTPγS binding assays were fit to a single exponential rate, whereas data for controls and the 3Nb GTPγS binding assay were fit to a double exponential model, due to the presence of a slow kinetic phase, and the kinetic constant of the fast rate (k1) reported. In all cases average and standard deviations of measurement are reported for a minimum of 5 experimental replicates. **b**, Sample progress curves for the data reported in panel **a.**

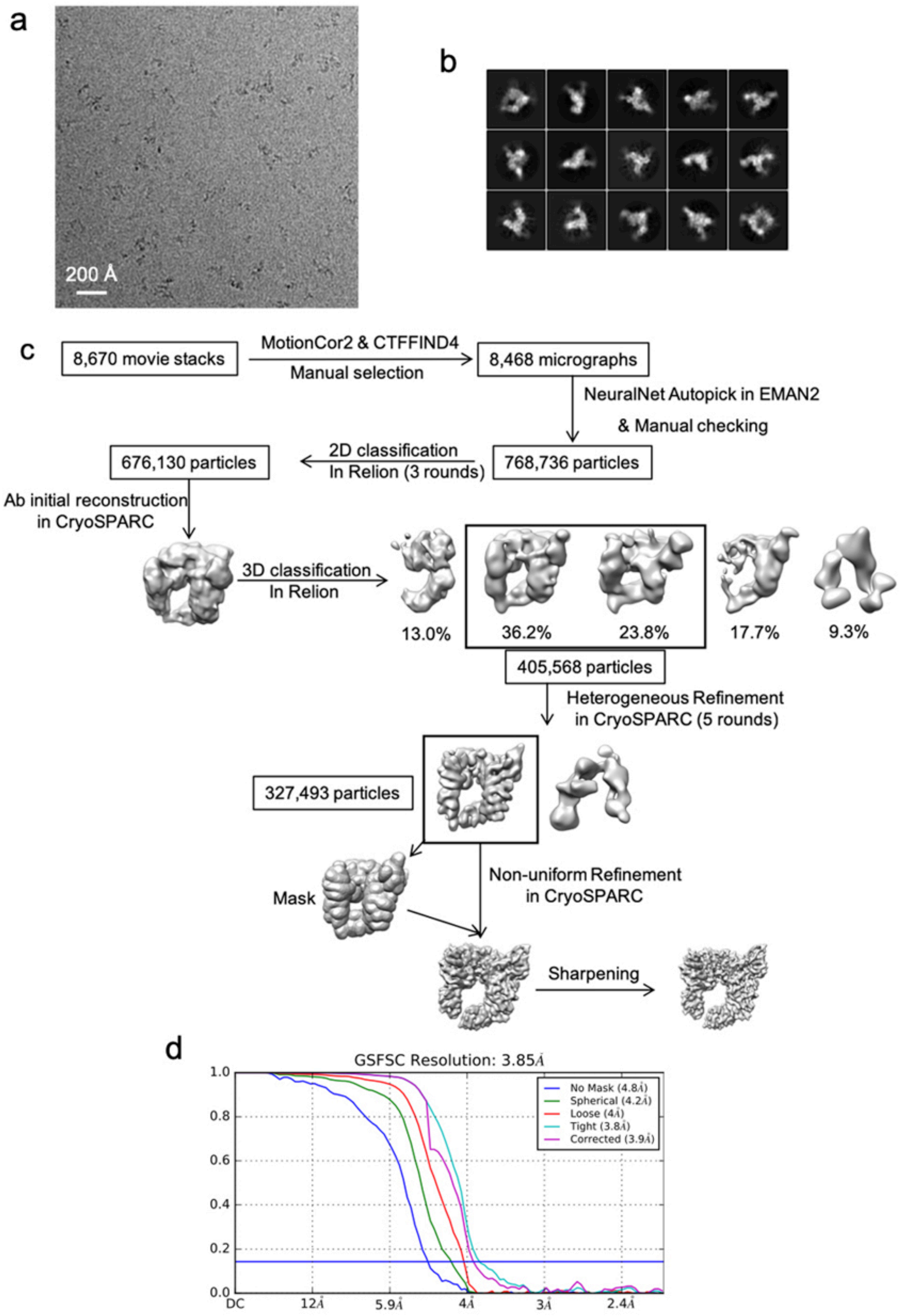

Supplementary Figure 3. Single-particle cryo-EM of Ric-8A:Gα:4Nb complex and Workflow of cryo-EM image processing. a, Representative motion corrected cryo-EM micrograph. **b**, Reference-free 2D Class averages computed in Relion. **c**, Workflow for cryo-EM processing (see Methods). **d**, Gold-standard Fourier Shell Correlation plot for 3D reconstruction generated using CryoSPARC.

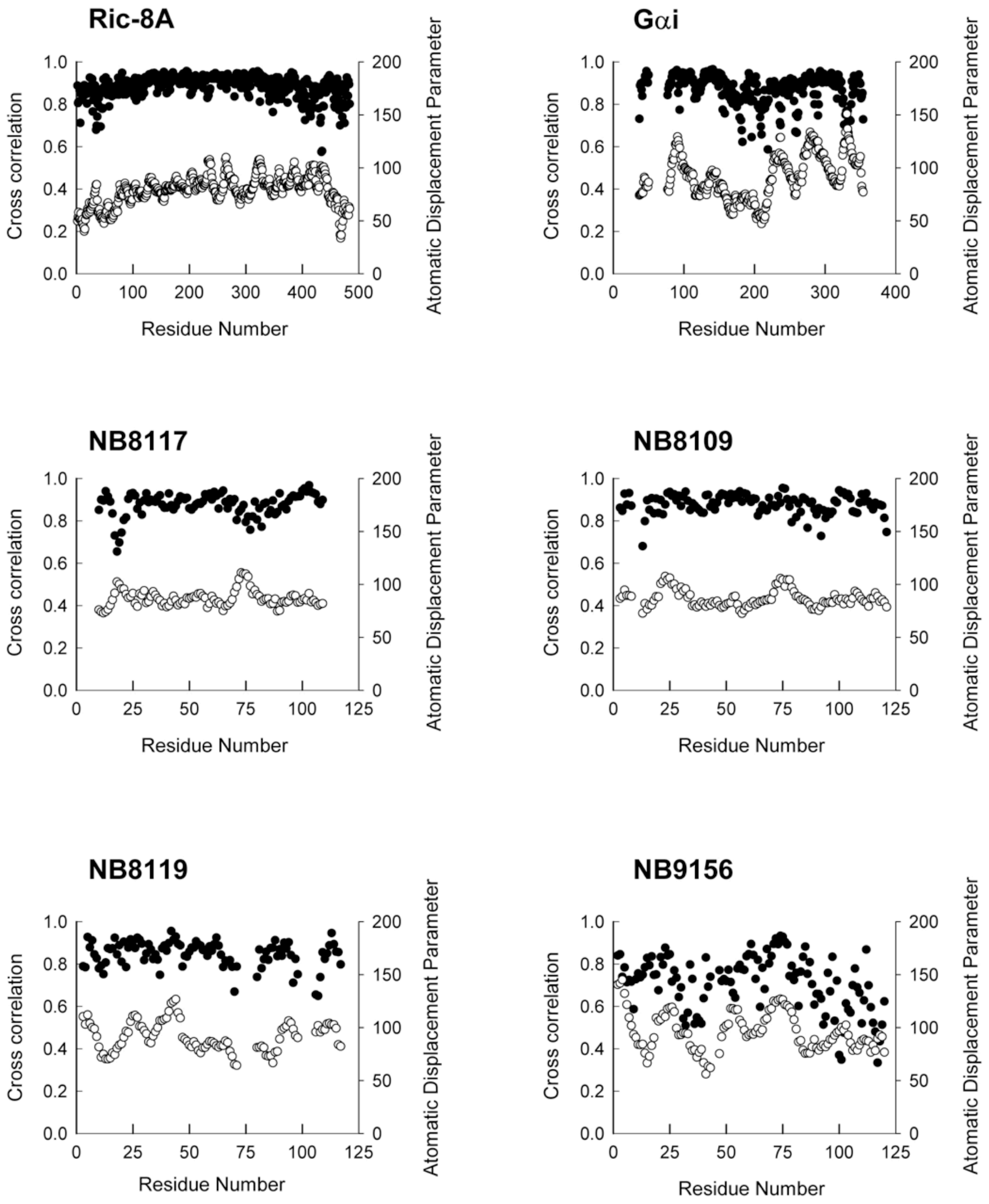

Supplementary Figure 4. Real-space refinement of Ric-8A:Gα model fit to cryo-EM map. Model-map correlation (filled circles) and atomic displacement parameter (open circles) are plotted with respect to residue number for the macromolecular components of the Ric-8A:Gα:4NB model derived from cryo-EM map. Data were generated using the phenix.real space refine program from the PHENIX suite.

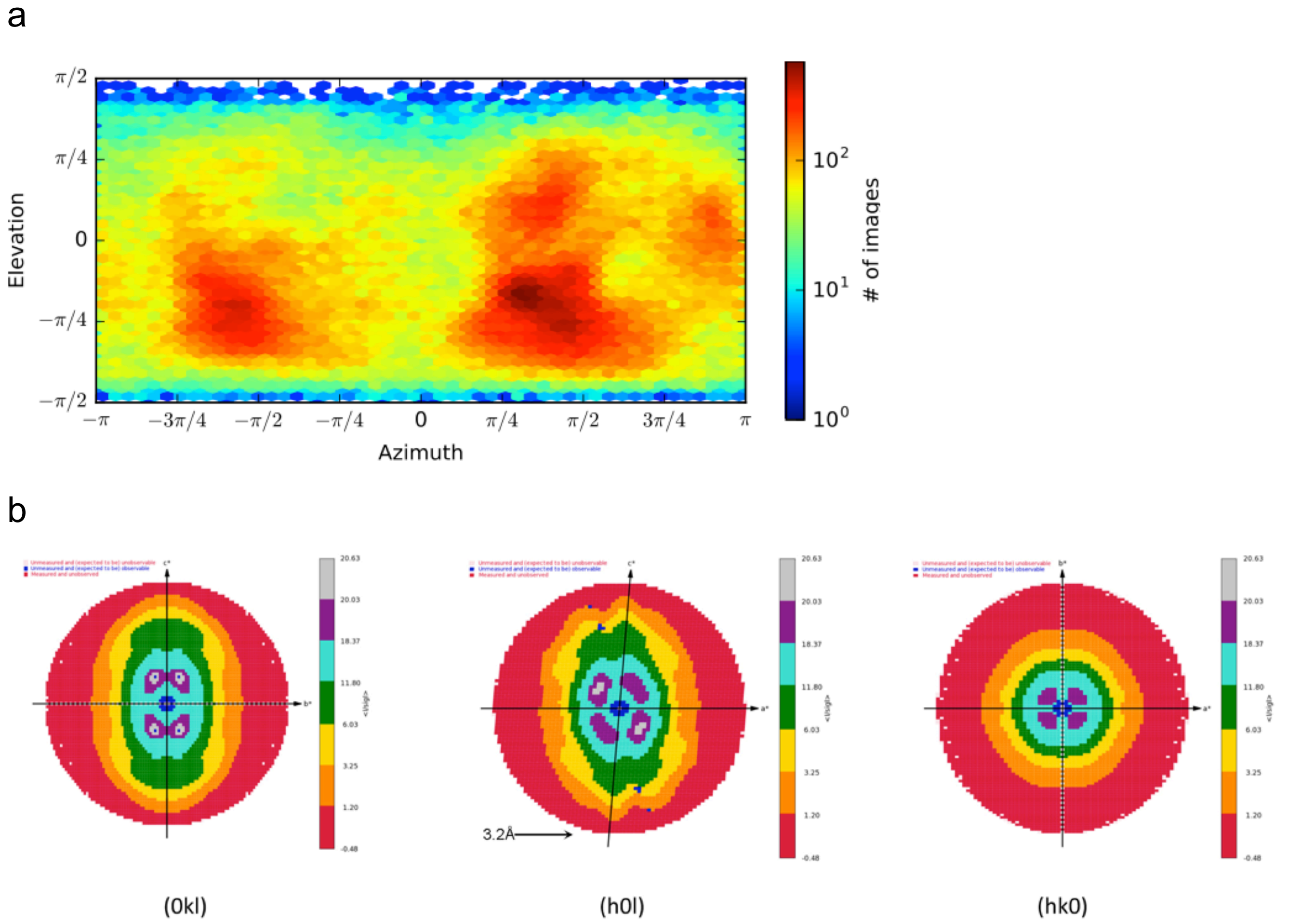

Supplementary Figure 5 Completeness of Cryo-EM and X-ray crystallographic data in real and reciprocal space. a, Distribution of particle orientations, generated using cryoSPARC in images used to generate 3D cryo-EM reconstruction. **b,** Projections of diffraction intensities from crystals of the Ric-8A:Gα:3Nb complex along reciprocal cell axes and color-coded by I/σI. Data generated using the STARANISO sever (see Methods).

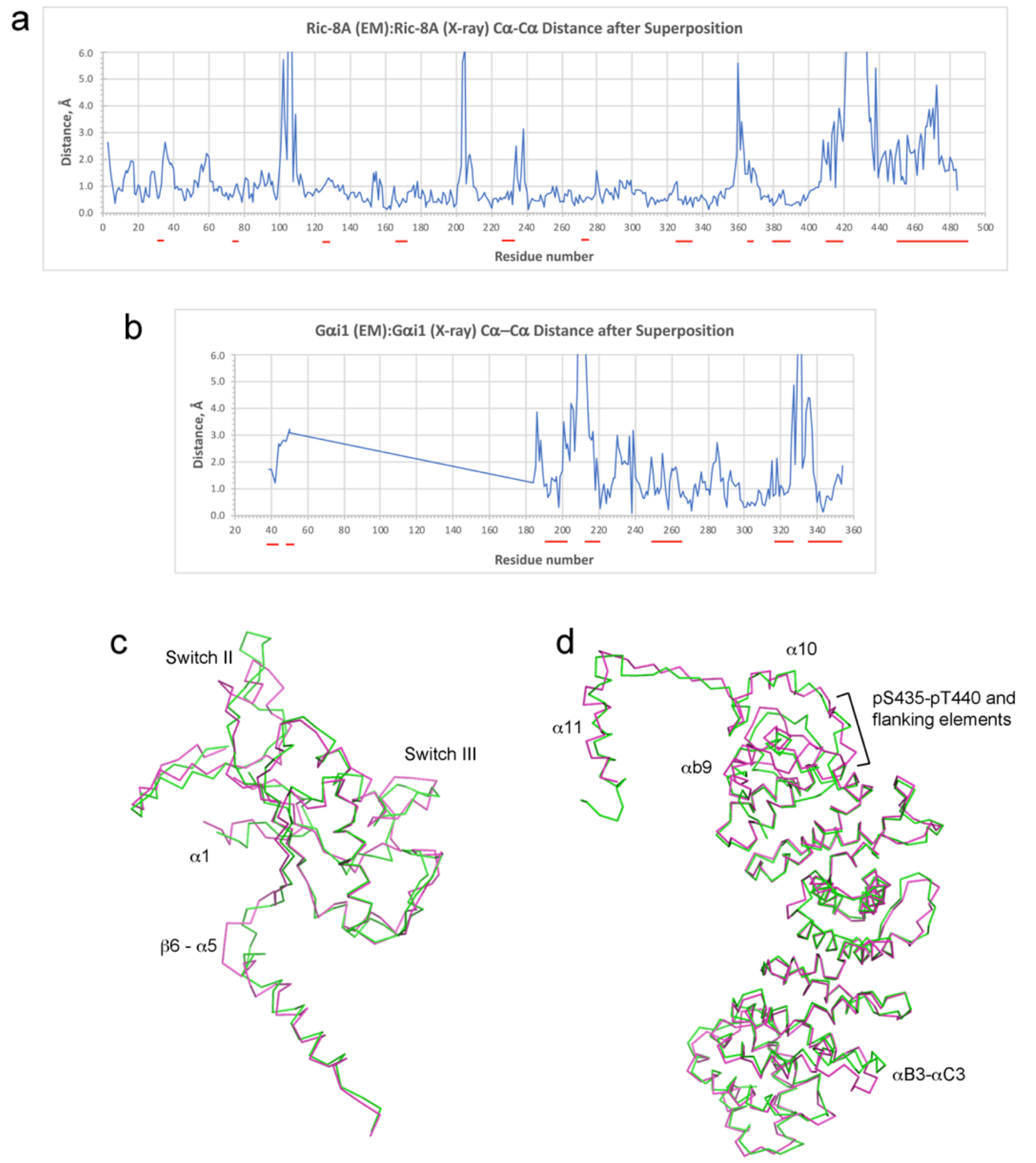

Supplementary Figure 6. Agreement of cryo-EM and X-ray structures of Ric-8A:Gαi1. a, Deviation between corresponding Cα atoms after least-squares superposition of Ric-8A coordinates. *Red* lines below residue numbers mark regions at which Ric-8A contacts Gai1 (see also ED Figure 10). The RMSD for all 484 common Cα positions is 1.81Å. The PyMOL align routine yields an RMSD of 1.03Å after rejection of 69 outliers. **b**, Deviation between Gαi1 atoms, computed per panel **a**. The RMSD for all 183 common Cα positions is 2.36Å; PyMol align yields an RMSD of 1.49Å after rejection of 21 outliers. **c,** Superposition of cryoEM (*magenta*) and X-ray (*green*) structures of Gαi1. Structural elements that show the largest structural differences are labeled. **d**, Superposition of Ric-8A models, as per panel **c.**

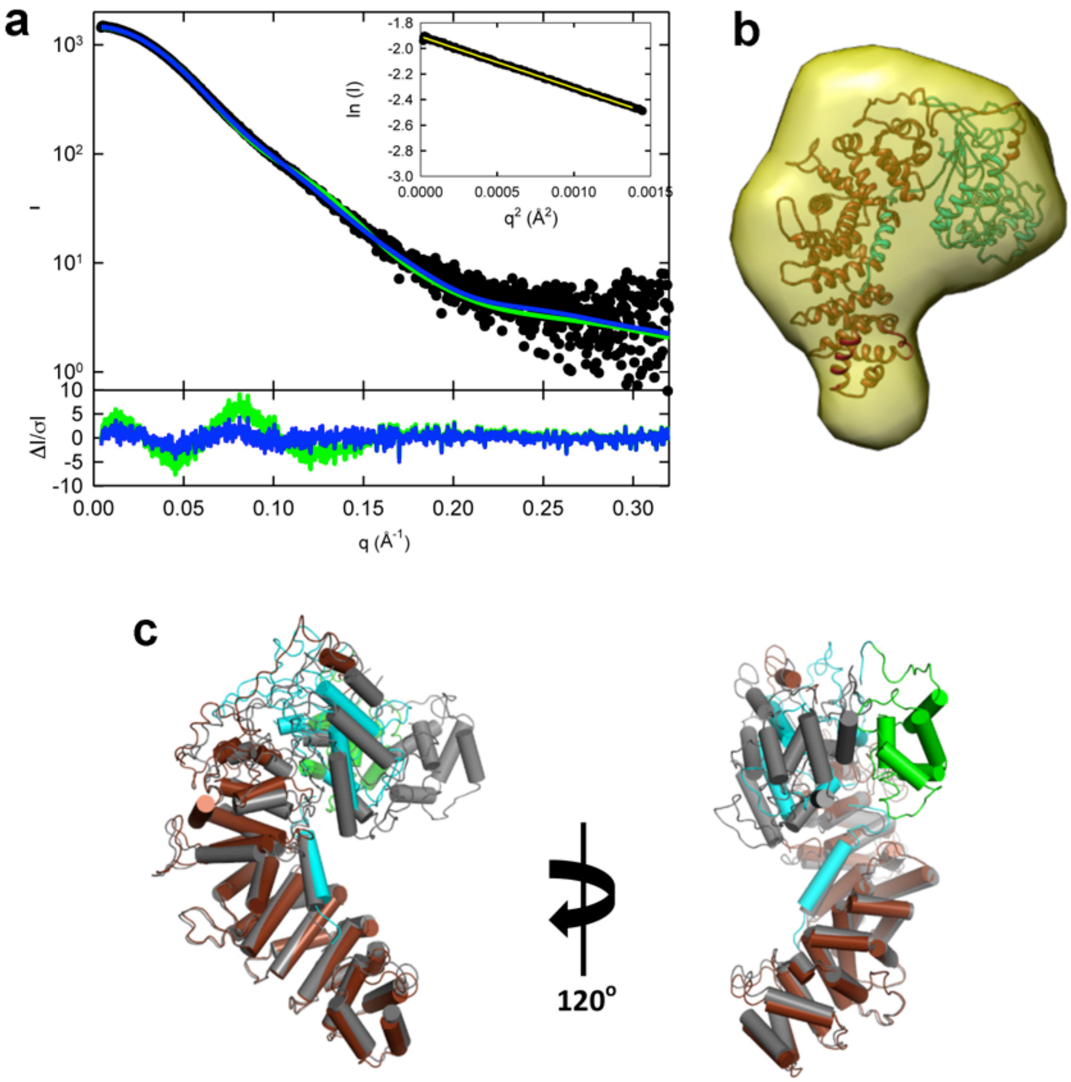

Supplementary Figure 7. Solution structure and Normal Mode refined model of Ric-8A:Δ31NGαi1 complex from Small-Angle X-ray scattering. **a**, Solution scattering profile for Ric-8A:ΔN31Gαi1 complex (*closed circles*). Inset shows, associate Guinier plot. CRYSOL and SRELEX from the ATSAS software package were used, respectively, to compute scattering profiles from atomic coordinates, and use normal-mode analysis to generate a refined atomic model. Computed scattering curves for cryo-EM-derived atomic coordinates of Ric-8A:ΔN31Gαi1 complex are shown in *green* and that from normal mode analysis is rendered in *blue*. The χ^2 for the fit of the computed scattering curve for the cryo-EM model is 5.48 and that for the normal-mode refined model is 1.24. The bottom panel shows the error-weighted residual difference plots $\Delta I/\sigma I = (I(q)_{experimental} - CI(q)_{model})/\sigma I(q)$ *vs q*, where C is a normalizing scale factor. **b,** SREFLEX-refined Ric-8A:ΔN31Gαi1 model fit to the *ab initio* molecular envelope calculated from the experimental SAXS scattering curve (*yellow*). **c**, Superposition of the SREFLEX-refined model of Ric-8A:ΔN31Gαi (Ric-8A, *brown*; GTPase domain of ΔN31GαI, *cyan*; Helical domain of ΔN31GαI, *green*) onto the cryo-EM model (*gray*), indicating a mode of helical domain rotation that may be preferred in solution in the absence of nanobodies. Models were superimposed using the Cα atomic positions of Ric-8A.

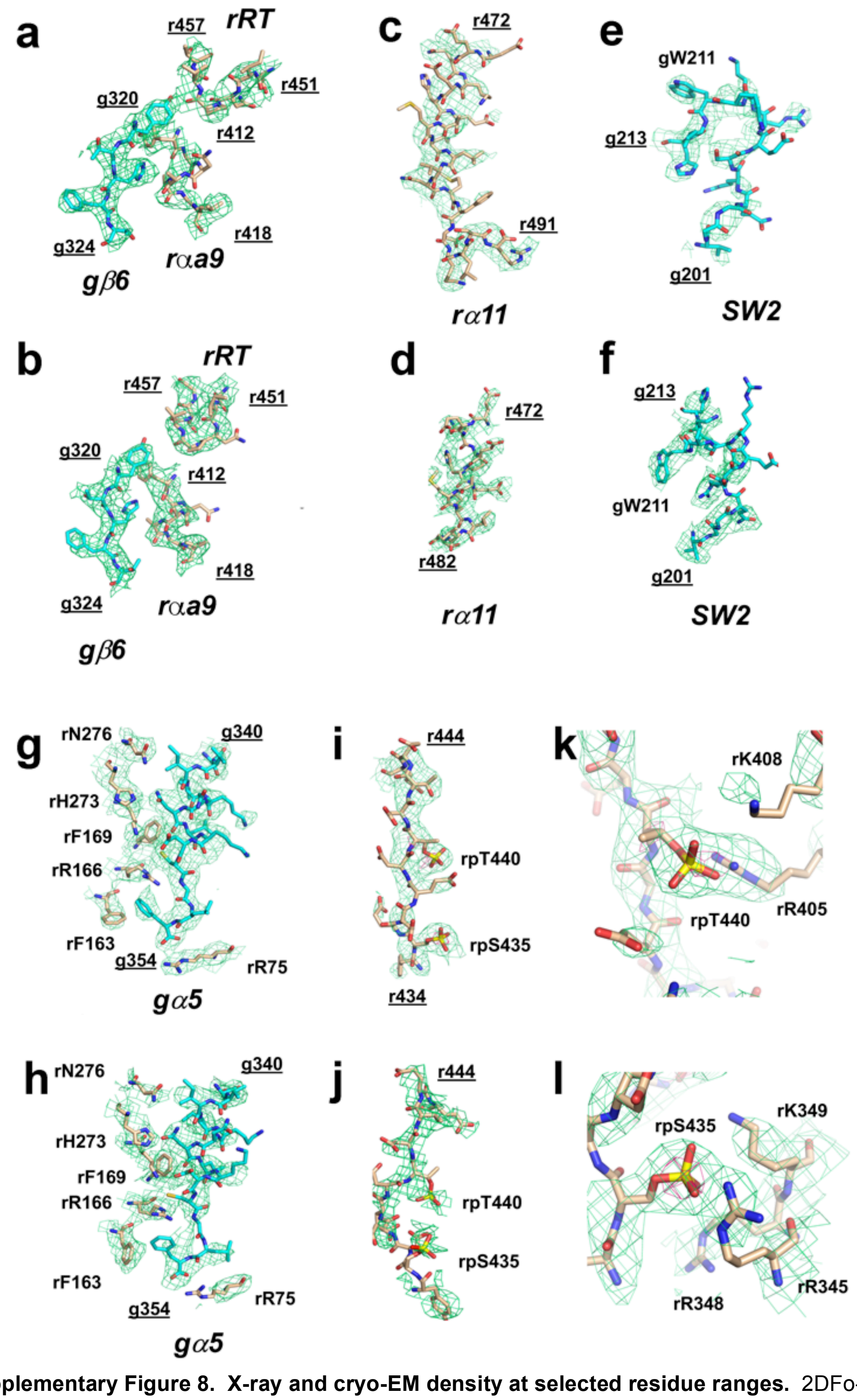

Supplementary Figure 8. X-ray and cryo-EM density at selected residue ranges. 2DFo-mFc electron density is contoured at 1.0σ and cryo-electron density is contoured at a threshold of 3.5. Structural elements of Ric-8A and Gαi1 are labeled in bold italics, N- and C-terminal residue numbers of segments are shown underlined, selected residues are labeled. Carbon atoms in Ric-8A are colored *wheat* and in Gαi1, *cyan*. Panels **a, c, e, g** and **i** show residues from crystallographic model with corresponding density. Panels **b, d, f, h** and **j** show the same from the cryo-EM model. Panels **k** and **l**, show the crystallographic model and associated density around phospho-serine 435 and phospho-threonine 440, respectively. The 5σ contour level is shown in *red*.

```
Ric8A_RAT       28 EHSQSFTF  71 RILSRDRSCL  121 LCNLVLSSPTAQ  160 VQFFDLRLLFLLTAL
Ric8A_MOUSE     28 EHSQSFTF  71 RILSRDRSCL  121 LCNLVLSSPTAQ  160 VQFFDLRLLFLLTAL
Ric8A_HUMAN     28 EHSQSFTF  71 RILSRDRNCL  121 LCNLVLSSPVAQ  160 VQFFDLRLLFLLTAL
Ric8A_BOVIN     28 ENSQSFTF  71 RILSRDRSCL  121 LCNLVLSSPVAQ  160 VQFFDLRLLFLLTAL
Ric8A_DANRE     28 EKSQCFSF  71 RILSRDKKSL  121 LCNIVFNSEAAQ  160 VRFFDLRLTFLITAL
Ric8A_CHICK     28 EKSQCFTF  71 RILSRDKYCL  121 LCNIVFSSPRAQ  160 VKFFDLRLLFLLTAL
Ric8A_XENTR     28 ENSQCFFF  71 RILSRDKHAL  122 LCNIIYNSVEAQ  161 SKFFDLRLLFLLTAL
Ric8B_DANRE     28 ENSRTFAF  71 RILSRDKHVL  127 LCNVVFNSVPAQ  164 VGLFSLRLLFLLSAL
Ric8B_XENTR     16 QNRTMFKF  59 RILSRDKKVL  109 LCNIVFNSMEAQ  148 IRCFALRLLFLLTLL
Ric8B_CHICK     30 ENRAVFRF  73 RILSRDKKVL  123 LCNIIFNSAVAQ  162 IKCFDLRLLFLLSLL
Ric8B_HUMAN     38 KHRATFKF  71 RILSRDKKVL  121 LCNIVFNSQMAQ  160 IKCFDLRLLFLLSLL
Ric8B_BOVIN     28 KHRATFKF  71 RILSRDKKVL  121 LCNIVFNSQMAQ  160 IKCFDLRLLFVLSLL
Ric8B_MOUSE     28 KHRATFKF  71 RILSRDKKIL  121 LCNIVFNSQMAQ  160 IKCFDLRLLFVLSLL
Ric8B_RAT       28 KHRATFKF  71 RILSRDKKIL  121 LCNIVFNSQVAQ  160 IKCFDLRLLFVLSLL
                    ::  -* *     ******:  *      ***::-.*  **        -* *** *::: *
                      αb1          αb2              αC3                αC4

Ric8A_RAT      225 KVLFNITFDSV  267 TEEFHGHTVNLLG  320 LHQTHRLKECVAPVLSVLTE  361 DVRTRPEVGDLLR
Ric8A_MOUSE    225 KVLFNITFDSV  267 TEEFHGHTVNLLG  320 LHQTHRLKECVAPVLNVLTE  361 DVRTRPEVGDLLR
Ric8A_HUMAN    226 KVLFNITLDSI  268 TEEFHGHAVNLLG  321 LHKTHRLKESVAPVLSVLTE  362 DVRTRPEVGEMLR
Ric8A_BOVIN    225 KVLFNITFDSI  267 TEELHGHAVNLLG  320 LHQTHRLKESVAPVLSVLTE  361 DVRTRPEVGELLR
Ric8A_DANRE    237 KILFNVTFDSN  280 TEEMHSHTVNLLG  333 LDRGNKLKETLLPSLNLLTE  374 DVKNRPEVGNALR
Ric8A_CHICK    233 KVLFNITFDSS  276 TEEFHSHTVNLLG  329 LDRGHKLKESLTPVLNLLTE  370 DVKNRPEVGNSLR
Ric8A_XENTR    233 KTLFNITFDIS  276 TEEFHGHTVNLLV  329 LDRGHKLRETLTPVLNLLTE  370 DVKNRPEVGNTLR
Ric8B_DANRE    237 KALFNLTMSDS  277 TEETHSHAINLLS  330 IDKGFNFKEGLTPVLSLLTE  371 DVKERPEIGDTIR
Ric8B_XENTR    220 KALFNVTLDSW  272 TEELHSNTVNLLS  315 IDKGCSYREGLTPVLSLLTE  356 DVSNRPEVGMTVR
Ric8B_CHICK    234 KALFNVTLDSW  276 TEELHSNAINLLS  330 IDKGSSYREGLTPVLSLLTE  361 DVSSRPEVGTTVR
Ric8B_HUMAN    232 KALFNVTVDSW  274 TEELHSNAVNLLS  353 IDKGSSYREGLTPVLSLLTE  304 DVTNRPEVGSTVR
Ric8B_BOVIN    232 KALFNVTVDSW  274 TEELHSNAVNLLS  353 IDKGSSYREGLTPVLSLLTE  304 DVTNRPEVGSTVR
Ric8B_MOUSE    232 KALFNVTVDSW  274 TEELHSNAVNLLS  353 IDKGSSYREGLTPVLSLLTE  304 DVTNRPEVGSTVR
Ric8B_RAT      232 KALFNVTVDSW  274 TEELHSNAVNLLS  353 IDKGSSYREGLTPVLSLLTE  304 DVTNRPEVGSTVR
                   * ***:*-.           *** *-:::***       :.:  - -* :-* *.:***       **  ***:*  :*
                        αC5                 αb6                      αC7                αA8

Ric8A_RAT      374 NKLVRLMTHLDTDVKRVAAEFLFVLCSESVPRFIKYTGYGNAAGLLAARGLMAGGRPEGQ 433
Ric8A_MOUSE    374 NKLVRLMTHLDTDVKRVAAEFLFVLCSESVPRFIKYTGYGNAAGLLAARGLMAGGRPEGQ 433
Ric8A_HUMAN    375 NKLVRLMTHLDTDVKRVAAEFLFVLCSESVPRFIKYTGYGNAAGLLAARGLMAGGRPEGQ 434
Ric8A_BOVIN    374 NKLVRLMTHLDTDVKRVAAEFLFVLCSESVPRFIKYTGYGNAAGLLAARGLMAGGRPEGQ 433
Ric8A_DANRE    387 NKLVRLMTHIDTDVKHCAAEFLFVLCKESVSRFIKYTGYGNAAGLLAARGLMRGGRDPGH 446
Ric8A_CHICK    383 NKLVRLMTHIDTDVKHCAAEFLFVLCKESVSRFVKYTGYGNAAGLLAARGLMAGGREEGE 442
Ric8A_XENTR    383 NKLVRLMTHVDTDVKHCAAEFLFVLCKENVSRFVKYTGYGNAAGLLAARGLMAGGRGEGR 442
Ric8B_DANRE    383 NKLVRLMTHVDMAVKQGAAEFLFVLCKESVDNLLKYTGYGNAAGLLMARGLLAGGRGETQ 443
Ric8B_XENTR    369 NKLVRLMTHVDLGVKQIAAEFLFVLCKERVDSLLKYTGYGNAAGLLAARGLLAGGRGDRW 428
Ric8B_CHICK    384 NKLVRLMTHVDLGVKQIAAEFLFVLCKERVDSLLKYTGYGNAAGLLAARGLLAGGRGDHW 443
Ric8B_HUMAN    407 NKLVRLMTHVDLGVKQIAAEFLFVLCKERVDSLLKYTGYGNAAGLLAARGLLAGGRGDNW 466
Ric8B_BOVIN    407 NKLVRLMTHVDLGVKQIAAEFLFVLCKERVDSLLKYTGYGNAAGLLAARGLLAGGRGDNW 466
Ric8B_MOUSE    407 NKLVRLMTHVDLGVKQIAAEFLFVLCKERVDSLLKYTGYGNAAGLLAARGLLAGGRGDNW 466
Ric8B_RAT      407 NKLVRLMTHVDLGVKQIAAEFLFVLCKERVDSLLKYTGYGNAAGLLAARGLLAGGRGDNW 466
                   *********:*--**:-*********.*  *-  ::*****************: ***
                        αB8             αC8       αa9          αb9

Ric8A_RAT      444 EYREAKASINPVTGRVEEKPPNPMEGMTEEQKEHEAMKLVNMFDKLSRHR 493
Ric8A_MOUSE    444 EYREAKASINPVTGRVEEKPPNPMEGMTEEQKEHEAMKLVNMFDKLSRHR 493
Ric8A_HUMAN    445 EYKEAKASINPVTGRVEEKPPNPMEGMTEEQKEHEAMKLVTMFDKLSRNR 494
Ric8A_BOVIN    444 EYKEAKASINPVTGRVEEKPPNPMEGMTEEQKEHEAMKLVNMFDKLSRHR 493
Ric8A_DANRE    457 EYREAKPHINPVTGRVEEEQPNPMEGMTDEQKEYEAMKLVNMFDKLSREQ 506
Ric8A_CHICK    453 EYKEAKPNINPVTGRVEEKLPNPMEGMTEEQKEYEAMKLVNMFDKLSREQ 502
Ric8A_XENTR    453 EYREAKPNINPVTGRVEEKQPNPMDGMTEEQKEYEAMKLVNMFDKLSREQ 502
Ric8B_DANRE    454 EYKSVKPFINPITGHVEDPLPNPLDEMTEEQKEYEAQRLVSMIDELSRKE 503
Ric8B_XENTR    439 EYLLAKPNINLITGHVEEPLPNPMDEMTEEQKEYEAMKLVNMFDKLSREE 488
Ric8B_CHICK    454 EYKSAKPNINLITGHLEEPMPNPMDEMTEEQKEYEAMKLVNMFDKLSRDE 503
Ric8B_HUMAN    477 EYKNAKPNINLITGHLEEPMPNPIDEMTEEQKEYEAMKLVNMLDKLSREE 526
Ric8B_BOVIN    477 EYKNAKPNINLITGHLEEPMPNPIDEMTEEQKEYEAMKLVNMLDKLSREE 526
Ric8B_MOUSE    477 EYKNAKPNINLITGHLEEPMPNPIDEMTEEQKEYEAMKLVNMLDKLSREE 526
Ric8B_RAT      477 EYKNAKPNINLITGHLEEPMPNPIDEMTEEQKEYEAMKLVNMLDKLSREE
                   **   .*  **-:**::*:-  ***::  **:****-**  :**.*:*:***..
                             RT                       α11
```

Supplementary Figure 9a. Amino acid sequences of Ric-8A and Ric-8B across phylogeny near Gα contact residues. Residue numbers are shown at the start and, for long tracts, the end of subsequences that harbor Ric-8A contacting-residues, highlighted in *green* in the sequence of rat Ric-8A, defined as those that include at least one atom within 4.0 of an atom in Gαi1. Amino acids not identical in rat Ric-8A and rat Ric-8B in *red* in the conservation summary at the bottom of each alignment. Central residues of secondary structural elements are shown below the conservation summary.

```
Gs    31 QKDKQVYRATHRLLLLGAGESGKSTIVKQM 60   212 FQVDKVNFHMFDVGGQRDERRKWIQCFNDVTA 243
Golf 102 REQKRDLQQTHRLLLLGAGESGKSTIVKQM 131  268 FQVDKVNFHMFDVGGQRDERRKWIQCFNDVTA 299
G12   46 ARERRAVRRLVKILLLGAGESGKSTFLKQM 75   214 FVIKKIPFKMVDVGGQRSQRQKWFQCFDGITS 245
G13   39 SREKTYVKRLVKILLLGAGESGKSTFLKQM 68   211 FEIKNVPFKMVDVGGQRSERKRWFECFDSVTS 242
GQ    30 NRDKRDARRELKLLLLGTGESGKSTFIKQM 59   194 FDLQSVIFRMVDVGGQRSERRKWIHCFENVTS 225
G11   30 ERDKRDARRELKLLLLGTGESGKSTFIKQM 59   194 FDLENIIFRMVDVGGQRSERRKWIHCFENVTS 225
GT3   24 QEDAERDARTVKLLLLGAGESGKSTIVKQM 53   189 FSFKDLNFRMFDVGGQRSERKKWIHCFEGVTC 220
GT1   20 KEDAEKDARTVKLLLLGAGESGKSTIVKQM 49   185 FSFKDLNFRMFDVGGQRSERKKWIHCFEGVTC 216
GT2   24 QEDADKEAKTVKLLLLGAGESGKSTIVKQM 53   189 FSVKDLNFRMFDVGGQRSERKKWIHCFEGVTC 220
GO    24 KEDGISAAKDVKLLLLGAGESGKSTIVKQM 53   190 FTFKNLHFRLFDVGGQRSERKKWIHCFEDVTA 221
GI2   24 REDGEKAAREVKLLLLGAGESGKSTIVKQM 53   190 FTFKDLHFKMFDVGGQRSERKKWIHCFEGVTA 221
GI1   24 REDGEKAAREVKLLLLGAGESGKSTIVKQM 53   189 FTFKDLHFKMFDVGGQRSERKKWIHCFEGVTA 220
GI3   24 REDGEKAAKEVKLLLLGAGESGKSTIVKQM 53   189 FTFKELYFKMFDVGGQRSERKKWIHCFEGVTA 220
         .:      - :****::*******::***          * ...: *::.******.:*::*:.**:.:*.
                   β1  P-loop   α1                β2    β3        Switch II

Gs   268 EALNLFKSIWNNRWLRTISVILFLNK 293  358 YCYPHFTCAVDTENIRRVFNDCRDIIQRMHLRQYELL 394
Golf 324 ESLDLFESIWNNRWLRTISIILFLNK 349  414 YCYPHFTCAVDTENIRRVFNDCRDIIQRMHLKQYELL 450
G12  270 ESMNIFETIVNNKLFFNVSIILFLNK 295  343 PLFHHFTTAIDTENIRFVFHAVKDTILQENLKDIMLQ 379
G13  267 ESLNIFETIVNNRVFSNVSIILFLNK 292  343 PLFHHFTTAIDTENIRFVFHAVKDTILQENLKDIMLQ 379
GQ   250 ESKALFRTIITYPWFQNSSVILFLNK 275  341 PLYHHFTTAINTENIRLVFRDVKDTILHDNLKQLMLQ 377
G11  250 ESKALFRTIITYPWFQHSSVILFLNK 275  323 IIYSHFTCATDTENIRFVFAAVKDTILQLNLKEYNLV 359
GT3  245 ESLHLFNSICNHKYFATTSIVLFLNK 270  318 EIYSHMTCATDTQNVKFVFDAVTDIIIKENLKDCGLF 354
GT1  241 ESLHLFNSICNHRYFATTSIVLFLNK 266  314 EIYSHMTCATDTQNVKFVFDAVTDIIIKENLKDCGLF 350
GT2  245 ESLHLFNSICNHKFFAATSIVLFLNK 270  318 EIYSHMTCATDTQNVKFVFDAVTDIIIKENLKDCGLF 354
GO   246 ESLMLFDSICNNKFFIDTSIILFLNK 271  318 EIYCHMTCATDTNNIQVVFDAVTDIIIANNLRGCGLF 354
GI2  246 ESMKLFDSICNNKWFTDTSIILFLNK 271  319 EIYTHFTCATDTKNVQFVFDAVTDVIIKNNLKDCGLF 355
GI1  245 ESMKLFDSICNNKWFTDTSIILFLNK 270  318 EIYTHFTCATDTKNVQFVFDAVTDVIIKNNLKDCGLF 354
GI3  245 ESMKLFDSICNNKWFTDTSIILFLNK 270  318 EIYTHFTCATDTKNVQFVFDAVTDVIIKNNLKDCGLF 354
         *:  :* :* . - : --*::*****          -  -*:* *-:*:*:: ** ---* *- :*:  --*-
           α3      β5        p1                 β6   p2              α5
```

Supplementary Figure 9b. Sequence of rat Gα isoforms within Ric-8A contact regions. Residue numbers are shown at the start and end of subsequences that harbor Ric-8A contacting-residues, highlighted in *green* in the sequence of Gαi1, defined as those that include at least one atom within 4.0 of an atom in Ric-8A. Amino acids not identical in Gαi and Gαs are highlighted in *red*, in the conservation summary at the bottom of each alignment. Central residues of secondary structural elements are shown below the conservation summary. Symbols p1 and p2 denote guanine nucleotide purine binding sites formed at the β5-αG and β6-α5 loops, respectively.

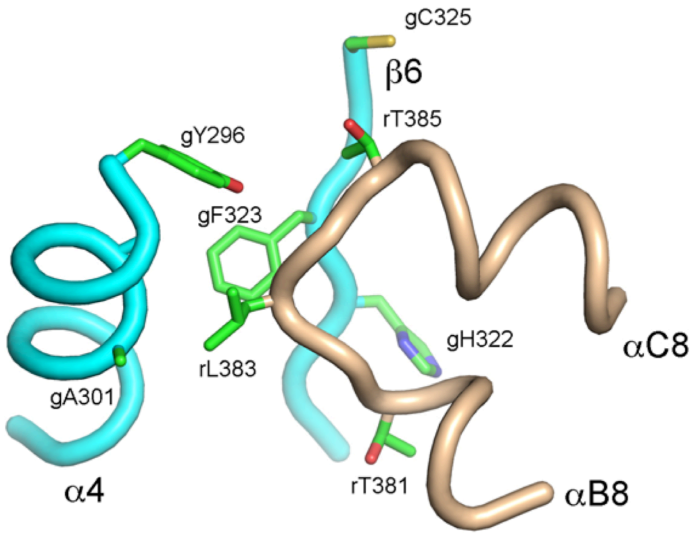

Supplementary Figure 10. Detail of Ric-8A:Gαi1 contact involving Gαi1 α4 and β6 with Ric-8A αB8-αC8. The crystal structure is shown with Gαi1 backbone in *cyan* and Ric-8A backbone in *wheat* with side-chain atom colors, carbon, *green*, nitrogen, *blue*, oxygen, *red* and sulfur, *yellow*. Mutation of rL383 to glutamate reduces GEF activity to 50% that of wild-type Ric-8A.

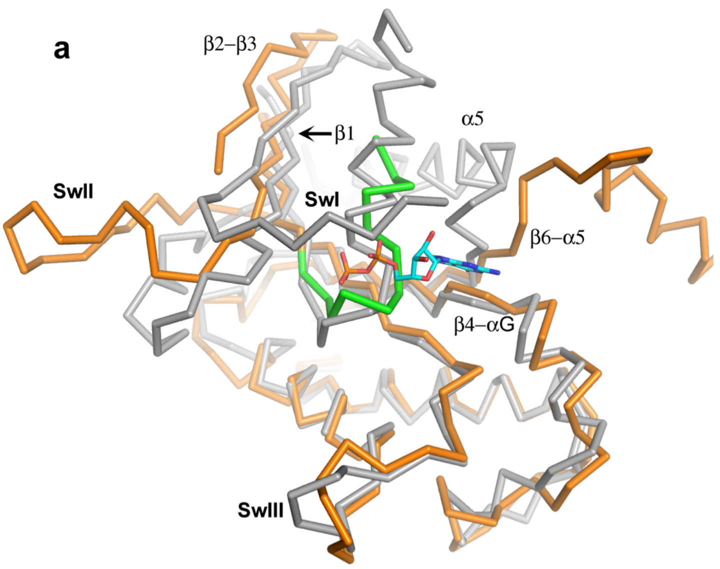

Supplementary Figure 11. Conformational perturbations induced in the Gα Ras domain by Ric-8A and a G protein-coupled Receptor. a, Cα-ribbon diagram showing superposition of Gαi1•GDP from the complex with Gβ1 and Gγ2 (PDB 1GG2), *gray*, onto the Ras domain of Gαi1, orange, bound to Ric-8A using residues 32-50 and 184-326. The α1 helix and P-loop of Ric-8A-bound Gαi1 is rendered *green*. GDP is shown as a stick model. **b**, Gαi1•GDP, as in panel **a** is shown superimposed, using the same residues, onto the Ras domain of Gαi2, magenta, bound to the A_1 Adenosine receptor (PDB 6D9H).
