# Supplementary material for: Structure of the G protein chaperone and guanine nucleotide exchange factor Ric-8A bound to Gαi1
Source: Nat Commun. 2020 Feb 26;11:1077. doi: 10.1038/s41467-020-14943-4 (PMC7044438; doi:10.1038/s41467-020-14943-4)
Supplement: Supplementary file 3 — Reporting Summary [file 41467_2020_14943_MOESM3_ESM.pdf]

nature research

Corresponding author(s): *Double-blind peer review submissions: write DBPR and your manuscript number here instead of author names.*

Last updated by author(s): *YYYY-MM-DD*

Reporting Summary

Nature Research wishes to improve the reproducibility of the work that we publish. This form provides structure for consistency and transparency in reporting. For further information on Nature Research policies, see Authors & Referees and the Editorial Policy Checklist .

Statistics

For all statistical analyses, confirm that the following items are present in the figure legend, table legend, main text, or Methods section.

n/a	Confirmed	
☐	☒	The exact sample size (n) for each experimental group/condition, given as a discrete number and unit of measurement
☐	☒	A statement on whether measurements were taken from distinct samples or whether the same sample was measured repeatedly
☐	☒	The statistical test(s) used AND whether they are one- or two-sided<br>*Only common tests should be described solely by name; describe more complex techniques in the Methods section.*
☒	☐	A description of all covariates tested
☒	☐	A description of any assumptions or corrections, such as tests of normality and adjustment for multiple comparisons
☐	☒	A full description of the statistical parameters including central tendency (e.g. means) or other basic estimates (e.g. regression coefficient) AND variation (e.g. standard deviation) or associated estimates of uncertainty (e.g. confidence intervals)
☐	☒	For null hypothesis testing, the test statistic (e.g. F, t, r) with confidence intervals, effect sizes, degrees of freedom and P value noted<br>*Give P values as exact values whenever suitable.*
☒	☐	For Bayesian analysis, information on the choice of priors and Markov chain Monte Carlo settings
☒	☐	For hierarchical and complex designs, identification of the appropriate level for tests and full reporting of outcomes
☒	☐	Estimates of effect sizes (e.g. Cohen's d, Pearson's r), indicating how they were calculated

Our web collection on statistics for biologists contains articles on many of the points above.

Software and code

Policy information about availability of computer code

Data collection	Cryospark<br>Relion
Data analysis	SigmaPlot7<br>AutoPROC v1.0.2 (XDS, and CCP4 programs SCALEA, TRUNCATE, POINTLESS and AIMLESS) are executed within AutoPROC)<br>PHENIX v1.1.6<br>Coot v0.8.6<br>MotiionCor2<br>EMAN v2.31<br>Relion v3.0.6<br>cryoSPARK v2<br>BioXTAS RAW v1.6.0<br>ATSAS v2.6.2 (calls DAMMIN, DAMMIF, DAMAVER, SREFLEX and CRYSOL)<br>Cluustal Omega<br>Molprobity<br>PDB Validation Report

For manuscripts utilizing custom algorithms or software that are central to the research but not yet described in published literature, software must be made available to editors/reviewers. We strongly encourage code deposition in a community repository (e.g. GitHub). See the Nature Research guidelines for submitting code & software for further information.

Data

Policy information about availability of data

All manuscripts must include a data availability statement. This statement should provide the following information, where applicable:

- Accession codes, unique identifiers, or web links for publicly available datasets
- A list of figures that have associated raw data
- A description of any restrictions on data availability

Coordinates of Ric-8A:Δ31Gαi1:4Nb model from cryo-EM are deposited in the RCSB Protein Data Bank (PDB) with ID 6UKT [http://dx.doi.org/10.2210/pdb6UKT/pdb]. Coordinates of Ric-8A:Δ31Gαi1:3Nb model from crystal structure are deposited in the PDB with ID 6YTL [http://dx.doi.org/10.2210/pdb6YTL/pdb]. The cryo-EM reconstruction is deposited in the Electron Microscopy Data Bank with id EMD20812 [https://www.ebi.ac.uk/pdbe/entry/emdb/EMD-20812]. The small angle X-ray scattering data for the Ric-8A:Δ31Gαi1 complex is deposited in the Small Angle Scattering Biological Database (SASBDB) with accession code SASDG95 [https://www.sasbdb.org/data/SASDFA5/]. The source data underlying Fig. 4 and Supplementary Fig 2a are provided as a Source Data file. All other relevant data are available from the authors.

Field-specific reporting

Please select the one below that is the best fit for your research. If you are not sure, read the appropriate sections before making your selection.

☒ Life sciences ☐ Behavioural & social sciences ☐ Ecological, evolutionary & environmental sciences

For a reference copy of the document with all sections, see nature.com/documents/nr-reporting-summary-flat.pdf

Life sciences study design

All studies must disclose on these points even when the disclosure is negative.

Sample size	For nucleotide exchange and GTP binding assays, progress curves were taken for a minimum of technical replicates for each Ric-8A mutant or nanobody condition (test of Ric-8A activity in the presence or absence of 3 or 4 nanobodies). Assays were conducted over a span of several days. To monitor any changes in Ric-8A activity during this period, additional replicates of selected mutants or nanobody conditions, which had been measured on previous days, were taken. Consequently, up to 10 replicates of any one mutant or nanobody condition.
Data exclusions	data were not rejected
Replication	all findings were replicated (see sample size)
Randomization	not applicable
Blinding	none

Reporting for specific materials, systems and methods

We require information from authors about some types of materials, experimental systems and methods used in many studies. Here, indicate whether each material, system or method listed is relevant to your study. If you are not sure if a list item applies to your research, read the appropriate section before selecting a response.

Materials & experimental systems

n/a	Involved in the study	
☒	☐	Antibodies
☒	☐	Eukaryotic cell lines
☒	☐	Palaeontology
☒	☐	Animals and other organisms
☒	☐	Human research participants
☒	☐	Clinical data

Methods

n/a	Involved in the study	
☒	☐	ChIP-seq
☒	☐	Flow cytometry
☒	☐	MRI-based neuroimaging
