# Supplementary material for: Structure of the G protein chaperone and guanine nucleotide exchange factor Ric-8A bound to Gαi1
Source: Nat Commun. 2020 Feb 26;11:1077. doi: 10.1038/s41467-020-14943-4 (PMC7044438; doi:10.1038/s41467-020-14943-4)
Supplement: Supplementary file 4 — Description of Additional Supplementary Files [file 41467_2020_14943_MOESM4_ESM.docx]

**Description of Additional Supplementary Files**

File name: Supplementary Movie 1
Description: Tour of Cryo-EM structure of the Ric-8A:G complex provides a dynamic view of the nanobody-bound complex with a secondary structure cartoon selected side chains superimposed on cryo-EM density. The tour visits structural features described in the text.

File name: Supplementary Movie 2
Description: Structural transitions from free Ric-8A and G i1:GDP (PDB 1GIT) to Ric-8A:G. Free Ric-8A is modeled with residues C-terminal to the reverse turn in an arbitrary extended conformation. G, and its molecular surface is colored: GTPase domain, gray; Helical domain, green; switch II, cyan and g 5, red.
