# Supplementary material for: Structure of the G protein chaperone and guanine nucleotide exchange factor Ric-8A bound to Gαi1
Source: Nat Commun. 2020 Feb 26;11:1077. doi: 10.1038/s41467-020-14943-4 (PMC7044438; doi:10.1038/s41467-020-14943-4)
Supplement: Supplementary file 2 — Peer Review [file 41467_2020_14943_MOESM2_ESM.pdf]

Reviewers' comments:

Reviewer #1 (Remarks to the Author):

This paper describes the structure of the heterotrimeric G protein Galphai (Gai) in complex with its chaperone, Ric-8A, which is important for Galpha subunit production in cells. The paper is unusually rigorous in that both X-ray and cryoEM structures are presented, with similar results from both approaches. The structures required the use of camelid nanobodies which undoubtedly helped with crystallization and particle alignment. The work provides a notable advance over prior work reported by ref. 18, which provided a structure of Ric8A in complex with a Galpha C-terminal helix, which is where most of the selectivity is mandated for Galpha isoforms. However, that prior structure could not have anticipated the intricate contacts made by the C-terminal regions of Ric-8A with the GTPase domain of Gai. In essence, these structures resolve how Galpha subunits are held in a GTP-binding competent conformation while preventing its aggregation. The paper also provides some complementary SAXS data and functional analysis of interesting contact results observed with the Ras-like domain of the alpha subunit, as well as an explanation of why phosphorylating of Ric-8A contributes to its efficacy. There is little to fuss about technically and while the primary results are not broadly extendable to other fields, it will be of strong interest to the broader GPCR community.

The write-up is excellent and does a good job linking these new results back to what is generally know about Galpha subunit structure and dynamics.

Editorial comments:

1) Looking over the Molprobity report, there are quite a few severe clashes listed at the head of the clash list. Do the authors feel like they they are simply at the limits of the resolution of their maps and this is the best representation? Or perhaps some work on the structure could be done to resolve the most severe steric problems?

2) It would be interesting to have a comparison between Gai bound to Ric-8A with Gai bound to a GPCR to see where commonalities and differences may be in terms of nucleotide destabilization mechanisms. A major conclusion of the b2AR-Gs complex work was that both alpha5 and beta1 of Galpha seemed to be playing a role in control of nucleotide affinity. The beta1 role is controversial. Is there evidence of beta1 involvement in the case of Ric-8A? I know the authors briefly compare in the text, but could be elaborated on.

3) I think a supplemental figure summarizing the particle orientations for the cryo-EM reconstruction would be a good addition.

4) The SAXS data is actually not really useful (sorry) and if space is an issue could be jettisoned.

5) Line 113. What is meant, exactly, by "irregularly structured"? If taken literally, it would mean that the amino acids in these regions are doing usual structural things, and I don't think that is what the authors are going for.

6) Line 227. "In GPCR complexes with heterotrimers, switch II is protected by Gbg." That's true, but what is not clear is if that is dependent on the nano body that spans the interface of Galpha the Gbg and stabilizes the entire complex for crystallographic analysis. It isn't clear to me what is really going on in this assembly in a more physiological context. Maybe more caution is warranted in this kind of comparison?

Reviewer #2 (Remarks to the Author):

The manuscript authored by McClelland et al. provides an elegant and detailed structural study of the interaction of Ric-8A with the heterotrimeric G protein subunit Gαi1. This work builds upon previous biophysical studies of Ric-8A/Gαi interactions previously published by the Sprang laboratory and provides additional details beyond those recently published in Nature Communications by Srivastava and Artemyev.

Specific comments.

1. The authors should provide a more detailed comparison between this study and that published in Nature Communications by Srivastava and Artemyev.
2. The authors should reference and discuss the more recent JBC paper from the same authors.
3. Ric-8A is also tyrosine phosphorylated on residues nearby to S440 and T445 based on mass spectroscopy data. Can the authors comment on the possible consequence of such phosphorylation?

NCOMMS-19-42369-T: Response to reviewers

We thank the reviewers for their comments, which have provided opportunities to expand on topics that were sparsely covered in the original manuscript. Our response to reviewer comments are shown in italics, below, and are highlighted in the text.

Reviewer 1:

1) Looking over the Molprobity report, there are quite a few severe clashes listed at the head of the clash list. Do the authors feel like they they are simply at the limits of the resolution of their maps and this is the best representation? Or perhaps some work on the structure could be done to resolve the most severe steric problems?

*We conducted more than 5 rebuilds of both the cryoEM and crystallographic models during the course of refinement and are confident that further refitting to electron density and subsequent real-space (EM) or the combination of real-space and reciprocal space refinement (X-ray) in Phenix will not yield substantial improvement. The steric conflicts noted by Reviewer 1 are almost entirely due to clashes between hydrogen atoms, which are computationally positioned with respect to the heteroatoms by MOLPROBITY. This prompted us to conduct three rounds of refinement of the crystallographic model to which riding hydrogen atoms were applied within Phenix, thus allowing hydrogen atoms to be accounted for in computation of stereochemical restraints during refinement. As a consequence both R(work) and R(free) increased by about 0.2-0.3, and the clash score was reduced to 9 conflicts per 1000 atoms. Several steric conflicts of similar magnitude to those noted by Reviewer 1 appeared in the model refined with riding hydrogen atoms, but at different residues than those observed in the original refined model. Examination of the offending residues in the 2mF(obs)-DF(calc) map revealed electron density distributions that likely forced side chain conformations that favored inter-hydrogen clashes. We think that we are indeed at the limits of resolution, exacerbated by the very high anisotropy of X-ray scattering and have chosen for this reason not to make further modifications of the model. To avoid misleading claims regarding resolution, we have replaced the phrase "*affording measurement of a 90% complete anisotropic dataset to 3.3 Å resolution" *to "*affording measurement of a 90% complete anisotropic dataset" *at line 91*

2) It would be interesting to have a comparison between Gai bound to Ric-8A with Gai bound to a GPCR to see where commonalities and differences may be in terms of nucleotide destabilization mechanisms. A major conclusion of the b2AR-Gs complex work was that both alpha5 and beta1 of Galpha seemed to be playing a role in control of nucleotide affinity. The beta1 role is controversial. Is there evidence of beta1 involvement in the case of Ric-8A? I know the authors briefly compare in the text, but could be elaborated on.

We have expanded our discussion of comparative conformational changes induced in Gα by GPCRs and Ric-8A, and have added supplementary Figure 11 to illustrate the

*points made in the expanded text (lines 237-250): "*Comparison of the Ric-8A:Gαi1 complex with that of the $G\alpha i2{:}\beta_1\gamma_2$ heterotrimer bound to the A_1 adenosine receptor (**Supplementary Figure 11)** shows that both GEFs induce conformational changes or disorder within the Gα P-loop and in gα1. Binding within the transmembrane cavity of GPCRs, the C-terminus of Gα (gα5) undergoes a 60° rotation and 5Å displacement[18], thereby inducing rearrangement of gβ6-gα5 and, to a lesser extent, gβ4-αG, both of which are purine recognition elements. The destabilization of gα1 by loss of contacts with gα5 is transmitted both to the P-loop and the hinge between the Ras and helical domains, permitting the release of contacts between the two[2]. By its wholesale ejection of gα5 from the Gα β-sheet and perturbation of the Gα β-sheet itself, which is not observed in interactions with GPCRs, Ric-8A produces the same outcome. Remarkably, the C-terminus of Gα, which tends to disorder, forms anchoring contacts with both GPCRs and with Ric-8A. In contrast, GPCRs do not induce reorientation of the gβ2-gβ3 β-hairpin, β1 or major structural changes in Switch II that are observed in the complex of Ric-8A with Gαi1."

*To better describe the effect of Ric-8A binding in the Gα beta sheet, we have added the sentence: "*As a unit, gβ1-gβ5 undergo a ~5° counter-clockwise rotation as viewed from the concave surface of the Gα β-sheet. Changes in the orientation of gβ1-gβ3, in particular result in destabilization and partially disordering gα1" *at line 155.*

3) I think a supplemental figure summarizing the particle orientations for the cryo-EM reconstruction would be a good addition.

A figure generated using CryoSPARC has been included as supplementary figure 5a.

4) The SAXS data is actually not really useful (sorry) and if space is an issue could be jettisoned.

We would like to retain this figure because it does confirm that the helical domain of Gα bound to Ric-8A is flexible in solution, even though it is ordered in the cryo-EM structure (likely due to steric constraints imposed by nanobodies).

5) Line 113. What is meant, exactly, by "irregularly structured"? If taken literally, it would mean that the amino acids in these regions are doing usual structural things, and I don't think that is what the authors are going for.

Agreed, this wording is confusing; the offending phrase has been removed (line 113)

6) Line 227. "In GPCR complexes with heterotrimers, switch II is protected by Gbg." That's true, but what is not clear is if that is dependent on the nano body that spans the interface of Galpha the Gbg and stabilizes the entire complex for crystallographic analysis. It isn't clear to me what is really going on in this assembly in a more physiological context. Maybe more caution is warranted in this kind of comparison?

We have eliminated this sentence.

Reviewer 2

1. The authors should provide a more detailed comparison between this study and that published in Nature Communications by Srivastava and Artemyev.

2. The authors should reference and discuss the more recent JBC paper from the same authors.

In lines 170-172, we have remarked on the similarity between the interaction between the transducin C-terminus with Ric-8A observed by Srivastava and Artemyev (Nature Communications, 2019) and that of Gαi1 with Ric-8A seen in our structures: "Indeed, after superposition of the respective Ric-8A models, the main- and side-chain atoms of the C-termii of transducin (residues X-Y) and Gαi1(338-354) align with an RMS deviation of 0.36Å." *We have included a reference to the more recent JBC paper by these authors in the introduction (line 60) and in lines 200-203, noted that:* "The ejection of gα5 from the Gαi1 β-sheet together with interactions between α11 and the Switch II and gα3 interfaces was recently deduced from steered molecular dynamics calculations consistent with small angle X-ray scattering and crosslinking data[22]."

3. Ric-8A is also tyrosine phosphorylated on residues nearby to S440 and T445 based on mass spectroscopy data. Can the authors comment on the possible consequence of such phosphorylation?

Because the reported tyrosine phosphorylation has not been unambiguously confirmed by mass spectroscopic data, we are reluctant to comment on its possible structural consequences. The location and conformation of Y434, the proposed phosphorylation site, would potentially allow interactions with a nearby arginine residue in Ric-8A, but also has the potential to induce repulsive electrostatic interactions with other residues. Thus, it does not seem wise to speculate in the absence of structural data.
